# ProAlanase is an Effective Alternative to Trypsin for Proteomics Applications and Disulfide Bond Mapping

**DOI:** 10.1074/mcp.TIR120.002129

**Published:** 2020-10-05

**Authors:** Diana Samodova, Christopher M. Hosfield, Christian N. Cramer, Maria V. Giuli, Enrico Cappellini, Giulia Franciosa, Michael M. Rosenblatt, Christian D. Kelstrup, Jesper V. Olsen

**Affiliations:** 1 Novo Nordisk Foundation Center for Protein Research, University of Copenhagen, Copenhagen, Denmark; 2 Promega Corporation, Madison, Wisconsin; 3 Novo Nordisk A/S, Måløv, Denmark; 4 Department of Molecular Medicine, Sapienza University of Rome, Rome, Italy; 5 Evolutionary Genomics SectionGlobe Institute, University of Copenhagen, Copenhagen, Denmark

**Keywords:** Proteases*, Phosphoproteins*, De novo sequencing, Disulfides*, Proteolysis*, Histones*, ProAlanase, Proline-rich Proteins, PTMs

## Abstract

Trypsin is the protease of choice in bottom-up proteomics. However, its application can be limited by the amino acid composition of target proteins and the pH of the digestion solution. In this study we characterize ProAlanase, a protease from the fungus *Aspergillus niger* that cleaves primarily on the C-terminal side of proline and alanine residues. ProAlanase achieves high proteolytic activity and specificity when digestion is carried out at acidic pH (1.5) for relatively short (2 h) time periods. To elucidate the potential of ProAlanase in proteomics applications, we conducted a series of investigations comprising comparative multi-enzymatic profiling of a human cell line proteome, histone PTM analysis, ancient bone protein identification, phosphosite mapping and *de novo* sequencing of a proline-rich protein and disulfide bond mapping in mAb. The results demonstrate that ProAlanase is highly suitable for proteomics analysis of the arginine- and lysine-rich histones, enabling high sequence coverage of multiple histone family members. It also facilitates an efficient digestion of bone collagen thanks to the cleavage at the C terminus of hydroxyproline which is highly prevalent in collagen. This allows to identify complementary proteins in ProAlanase- and trypsin-digested ancient bone samples, as well as to increase sequence coverage of noncollagenous proteins. Moreover, digestion with ProAlanase improves protein sequence coverage and phosphosite localization for the proline-rich protein Notch3 intracellular domain (N3ICD). Furthermore, we achieve a nearly complete coverage of N3ICD protein by *de novo* sequencing using the combination of ProAlanase and tryptic peptides. Finally, we demonstrate that ProAlanase is efficient in disulfide bond mapping, showing high coverage of disulfide-containing regions in a nonreduced mAb.

## Abstract (precis)

The ProAlanase protease cleaves after proline and alanine residues in highly acidic conditions and provides efficient digestion of proline-rich proteins allowing for accurate phosphorylation site profiling. Because of its activity at low pH, it facilitates efficient disulfide bond mapping in monoclonal antibodies and is highly suitable for analysis of histone family members and their PTMs. The protease is complementary to trypsin and allows for increased protein sequence coverage and nearly complete *de novo* sequencing of exogenously expressed proteins when combined with trypsin.

## Graphical Abstract

**Figure:**
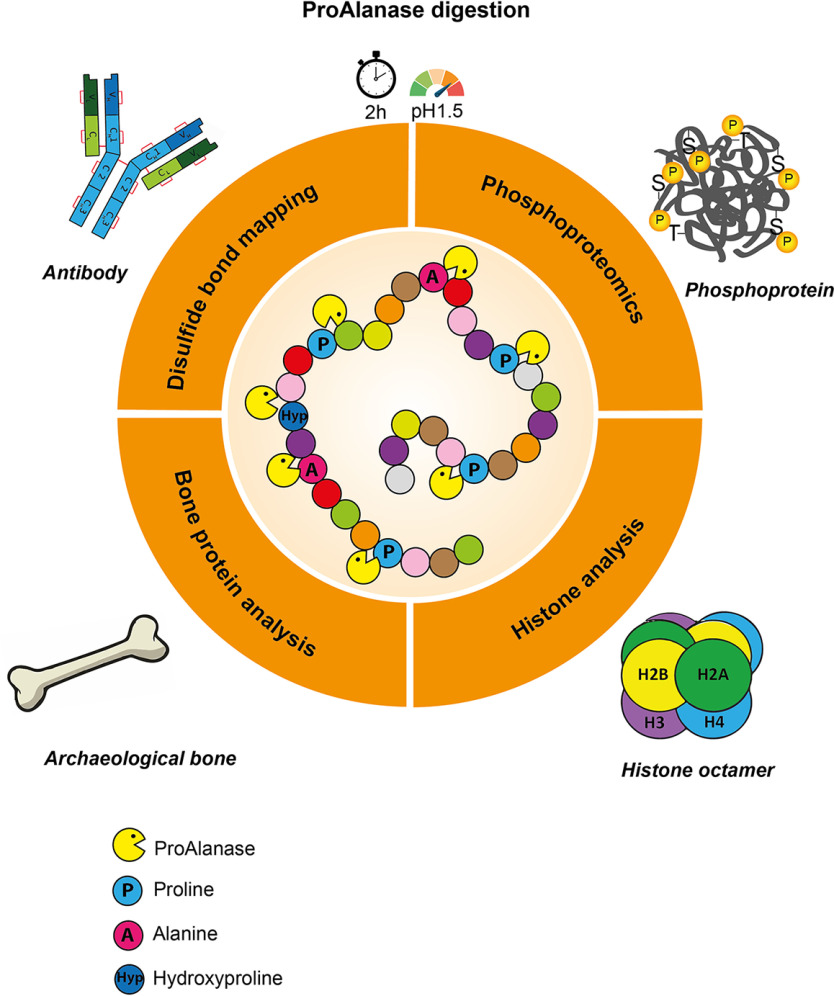

**Highlights**

- ProAlanase is a powerful protease for efficient low pH disulfide bond mapping.
- High suitability for analysis of histone family members and their PTMs.
- Accurate phosphorylation profiling in proline-rich proteins.
- Sequence coverage increase and full *de novo* sequencing in combination with trypsin.

In most proteomics investigations, proteins are enzymatically digested into shorter peptides, which are more amenable for sequencing by tandem MS (MS/MS) and identification via peptide-spectrum matching (PSM) (1). This digestion is typically conducted using a sequence-specific protease that cuts proteins after or before specific amino acids. Cleavage specificity, in turn, determines the precursor charge and peptide length distribution, as well as charge localization within the sequence. Trypsin is the most widely used protease in proteomics (2, 3). Sequencing grade trypsin is readily available, stable, and extremely specific with cleavage exclusively after the basic amino acids, arginine (R) and lysine (K) under normal experimental conditions (4). Because of the intermediary content of lysine and arginine in most proteins, tryptic peptides are typically relatively short yet unique enough to carry sequence information. Electrospray ionization generates mostly doubly-charged precursors containing a positively-charged arginine or lysine in their C terminus and an N-terminal positive charge on its α-amine group. These characteristics make tryptic peptides ideally suited for separation by reversed-phase liquid chromatography and they generally produce sequence-informative fragment MS/MS spectra by collision-induced dissociation (CID) dominated by y-type fragment ion series (2, 5). However, it is widely known that not all tryptic peptides are easy to analyze by MS. For example, KR-rich or KR-poor regions in proteins can lead to very short or very long tryptic peptides, which are difficult to analyze by standard reversed-phase LC–MS (RP-LC–MS/MS) (6, 7). In fact, 56% of all theoretical tryptic peptides derived from the human proteome are shorter than 7 amino acids and often cannot be used to uniquely identify specific proteins via MS (MS) (8). For example, the histone protein super family is characterized by a high content of lysine and arginine residues (9). Histones are the most abundant proteins in the eukaryotic cell nucleus, where they associate to DNA in a complex known as chromatin (10). Eukaryotic gene expression is tightly regulated by various covalent, site-specific PTMs (11) on histones, known as the histone code (12), which are proposed to affect transcription and chromatin remodeling processes. Consequently, analysis of histone modifications is a very important area of research with a growing community.

On the other hand, one example of KR-poor region is represented by the collagen protein family (13) whose proteins are mainly composed of the amino acids glycine (G), proline (P) and hydroxyproline (Hyp). These amino acids make up a characteristic repeating triplet motif, which ensures resistance of collagen toward proteolytic digestion. Nevertheless, there is a group of enzymes called “collagenases,” which degrade native collagen fibrils (14–16). These enzymes, however, often cause a reduction of bone proteome complexity alongside with collagen depletion (15), thus alternative proteases should be sought after (15).

Furthermore, although trypsin is mainly active in the pH range from 7 to 9 (17), in some cases lower pH digestion is required. An example of this is disulfide bond mapping, where sample preparation conditions with pH > 6 promote disulfide bond scrambling, which can result in the reconfiguration of disulfide bonds (18). Performing protein digestion with proteases that are active at acidic pH < 2 (18) eliminates disulfide bond scrambling by keeping free thiols fully protonated (19). Likewise, in hydrogen-deuterium exchange experiments, backbone amide hydrogen atoms of proteins may back-exchange. The minimum exchange rate occurs at approximately pH 2.5-2.6 (20). Therefore, to minimize the back-exchange, proteolysis and subsequent MS analysis must be performed at low pH (20).

One more challenge with trypsin is the cleavage of phosphopeptides containing basic phosphorylation sites, for example, (R or K)*n*(X)(S or T), which can result in ambiguous phosphosite localization (21). It is known that site-specific phosphorylation can rapidly modulate the function of target proteins by changing their enzymatic activity, subcellular localization, turnover and interaction partners (22). Hence, precise mapping of phosphorylation sites is important.

In bottom-up proteomics database search strategies are typically employed to identify proteins from complex mixtures (23). However, in some cases reference databases are not available. To overcome this issue, a database-independent sequence reconstruction (*de novo*) approach can be used to deduce peptide sequences directly from high-quality MS/MS spectra (24). Determination of a protein sequence without a reference database is highly relevant for analysis of proteins, derived from unsequenced or extinct organisms, environmental samples, and microbiomes. Other important examples are therapeutic antibodies and T-cell receptors for which the variable region sequences are unknown (25). Finally, *de novo* sequencing can be applied to analysis of bioactive bacterial or fungal peptides, containing nonproteinic or modified amino acids (26). To perform an efficient *de novo* protein sequencing by tandem MS, generation and analysis of overlapping peptides is typically required. This can be done by either conducting a multi-enzymatic protein digestion (3 - 4 proteases) and/or by applying orthogonal MS fragmentation techniques (27), meaning that tryptic digestion alone is not sufficient for *de novo* sequencing of the proteins.

Like trypsin, most of the commonly used proteases in proteomics, such as Lys-C, Lys-N, Arg-C, Asp-N and Glu-C, also cleave proteins at charged amino acids (6). Proteases with unique cleavage specificity may have significant value for proteomics because the resultant peptides are likely to come from different regions in the proteome. In 2009, an acidic prolyl-endopeptidase An-PEP from *Aspergillus niger* fungi was tested by Šebela *et al.* for possible proteomic applications, showing potential for in-solution protein digestion (28). An-PEP has also been tested in hydrogen-deuterium exchange experiments and protein structural analysis in 2017 and showed high suitability in that application (29). In 2019, the An-PEP protease, also named EndoPro, was characterized by Laarse *et al.* (30) and showed both complementarity to trypsin and high efficiency in phosphorylation profiling in selectively enriched phosphorylated peptides.

Here, we further characterize the protease and demonstrate its versatility in a wide variety of proteomics applications. Based on the observed proline- and alanine-directed specificity (∼80%), we call the protease ProAlanase, which more accurately reflects the proteolytic specificity. ProAlanase activity was found to be optimal for LC–MS applications when digestion times are kept short (2h) and pH is highly acidic (1.5). We used the optimized digestion conditions and applied them toward several relevant case studies including accurate phosphosite mapping and *de novo* sequencing of affinity purified exogenously expressed proline-rich protein. We also used ProAlanase to improve noncollagenous protein identification in archaeological bone and to increase histone sequence and PTM coverage in human proteome samples. Finally, ProAlanase digestion was successfully employed for low-pH disulfide bond mapping in a mAb. Taken together, these findings demonstrate the broad use of this protease for numerous proteomics applications.

## EXPERIMENTAL PROCEDURES

### 

#### 

##### Chemicals and Reagents

Chemicals were purchased from Sigma-Aldrich (Søborg, Denmark) unless otherwise specified. NIST mAb (mAb) was obtained from the National Institute of Standards and Technology (Gaithersburg, MD).

##### Human Cell Culture

Human epithelial cervix carcinoma HeLa cells (female origin) and human embryonic kidney HEK293T cells (female origin) were purchased from ATCC. Cells were cultured in DMEM high glucose with Glutamax (Invitrogen, 31966-021) with 10% fetal bovine serum (Invitrogen, 10270-106) and 100 U/ml penicillin/streptomycin (Invitrogen, 15140-122) at 37 °C in a humidified incubator with 5% CO_2_. Cell lines have been authenticated by STR profiling (ATCC, 135-XV) and periodically tested for mycoplasma via PCR (EZ-PCR Mycoplasma Test Kit, 20-700-20, Biological Industries).

##### HEK293T Cell Transfection

Human embryonic kidney HEK293T cells were transfected using LipofectAMINE 2000 (11668-019 – Invitrogen, Carlsbad, CA) with the plasmid pcDNA3-mN3ICD-FLAG, kindly provided by Dr. Isabella Screpanti (Sapienza University of Rome) (31). The used plasmid encodes the intracellular domain of murine Notch3 receptor which is N-terminally linked to the FLAG tag.

##### Cell Lysis

HeLa cells were harvested at approximately 90% confluency by washing twice with PBS (Invitrogen, 20012-068) and then adding 99 °C hot GndCl lysis buffer [6M guanidine hydrochloride (GndHCl), Sigma-Aldrich, G3272; 5 mm tris(2-carboxyethyl)phosphine (TCEP), Sigma-Aldrich, C4706–10G; 10 mm chloroacetamide (CAA); 100 mm Tris, pH 8.5, SigmaAldrich, 10708976001]. After rocking for 5 min, cells were scraped and lysate was boiled for 10 min at 99 °C, under 850 rpm agitation. DNA was sheared by 2 min ultrasonication treatment (Sonics & Materials, VCX 130; 1s on, 1s off, 80% amplitude).

HEK293T cells were lysed in ice-cold ImmunoPrecipitation lysis buffer [50 mm TrisHCl, pH 7.6; 150 mm Sodium chloride; 1 mm Calcium chloride; 1% Triton; 2 mm Sodium ortho-vanadate; Complete mini, EDTA-free; 5 mm Sodium fluoride; 5 mm Beta-Glycerol 2-phosphate]. After 30 min of ice-incubation, the lysates were centrifuged at 13000 rpm for 15 min and the supernatant was collected.

##### FLAG Immunoprecipitation

FLAG immunoprecipitation was performed using the anti-FLAG M2 affinity gel (A2220 – Sigma-Aldrich) according to manufacturer's instructions.

The samples were reduced with 10 mm TCEP, alkylated with 20 mm CAA and boiled for 10 min at 99 °C under 750 rpm agitation, before loading on SDS-PAGE gels. The gels were stained with Colloidal Blue Staining Kit (Invitrogen, LC6025) according to manufacturer's instructions. After the staining, the bands corresponding to Notch3 intracellular domain FLAG tag (mN3ICD-FLAG) molecular weight were excised, destained and further in-gel digested.

##### Pleistocene Mammoth Bone Protein Extraction and Manual Fractionation

Ancient protein extractions took place in facilities at the Natural History Museum of Denmark dedicated to the extraction of ancient DNA and ancient proteins. These include clean rooms fitted with filtered ventilation and positive air pressure, including additional measures recommended for ancient protein analysis (32). A negative extraction blank was processed alongside the ancient sample extractions, with the additional inclusion of injection blanks during MS/MS analysis to monitor potential protein contamination during all stages of analysis. Approximately 1 g of compact bone was crushed into a rough powder. Ancient protein residues were extracted from 20 mg of mineralized material using 300 µL 5.5% (1.8 M) hydrochloric acid (HCl) (Merck, CAS no. 7647-01-0) at 24 °C under intense agitation (850 rpm), overnight. After this, solubilized protein residues were centrifuged at maximum speed for 10 min and washed with 300 µL of MiliQ water, the procedure was repeated twice. The purified pellet was subsequently subject to protein extraction in hot extraction buffer [6M GndHCl, Sigma-Aldrich, G3272–2KG; 5 mm TCEP, Sigma-Aldrich, C4706–10G; 10 mm CAA; 100 mm Tris, pH 8.5, SigmaAldrich, 10708976001] at 80 °C for 1 h, under 850 rpm agitation. After 1 h of protein extraction, the samples were centrifuged in a benchtop centrifuge for 5 min at maximum speed resulting in 3 fractions (the pellet, the supernatant and pellet+supernatant), which were collected separately.

##### In-Solution and Protein Aggregation Capture Digestion of HeLa

Protein concentration was estimated by BCA assay (Thermo Fisher Scientific, 23225). Samples were diluted to approx. 0.3 M GndCl final concentration and 12 µg of protein per condition were digested with either 1:50 w/w ProAlanase prototype (Promega) for 2 h at pH 1.5, 37 °C or with Trypsin (Sigma-Aldrich), rAsp-N (Promega, VA1160) and Glu-C (Sigma-Aldrich) for 18 h at pH 8.5, 37 °C. After spinning down for 5 min at 17000 × *g*, peptides were immobilized and purified on an in-house packed StageTip C_18_ cartridges. Peptides were purified by three consecutive washes with 0.1% formic acid (FA), eluted in 40% acetonitrile (ACN) and evaporated in a SpeedVac^TM^ Concentrator (Thermo Fisher Scientific, Denmark) until ∼ 30 μl final volume. Peptide concentration was determined with a NanoDrop spectrophotometer (Thermo, Wilmington, DE) and samples were diluted to 0.1 µg/µL concentration with 0.1% trifluoroacetic acid (TFA) in 5% ACN. For protein aggregation capture (PAC) digestion (33), proteins from HeLa cell lysate were aggregated on magnetic micro-beads (acid-resistant hydroxyl-modified MagResyn bead prototype from Resyn Biosciences) by adding neat acetonitrile to 70% final concentration. Beads-to-protein ratio was 5:1. All subsequent washes were performed on a magnetic separation rack, and “on-bead” digestion with ProAlanase was performed for 2 h at pH 1.5, 37 °C. The resulting peptide mixtures were desalted and concentrated as described above.

##### In-Solution Digestion of K562

10 µg/µL MS-compatible K562 cell lysate (Promega, V6941) in 8 M urea was reduced by addition of 1 µL of 0.5 M DTT **(**DTT) (Sigma Aldrich, CAS no. 3483-12-3) to achieve a final concentration of 5 mm, and incubated at 37 °C for 30 min. The reduced lysate was alkylated by addition of 1.5 µL of 1 M iodoacetamide (IAA) (Sigma Aldrich, CAS no. 144-48-9) to achieve a final concentration of 15 mm, and incubated in the dark at room temperature for 1h. Reduced and alkylated cell lysate was diluted to approx. 0.3 M urea final concentration and 12 µg of protein were digested with 1:50 w/w ProAlanase prototype (Promega) for 2 h at pH 1.5, 37 °C. After spinning down for 5 min at 17000 × *g*, peptides were immobilized and purified on an in-house packed StageTip C_18_ cartridges. Peptides were purified by three consecutive washes with 0.1% formic acid (FA), eluted in 40% acetonitrile (ACN) and evaporated in a SpeedVac^TM^ Concentrator (Thermo Fisher Scientific, Denmark) until ∼ 30 μl final volume. Peptide concentration was determined with a NanoDrop spectrophotometer (Thermo, Wilmington, DE) and samples were diluted to 0.1 µg/µL concentration with 0.1% trifluoroacetic acid (TFA) in 5% ACN.

##### In-Gel Digestion of N3ICD

After destaining, protein concentration was estimated using BCA assay (Thermo Fisher Scientific, 23225) and samples were in-gel digested with either ProAlanase prototype [1:50 (w/w), HCl pH 1.5, 2 h digestion at 37 °C] or with Trypsin (Sigma-Aldrich), rAsp-N (Promega, VA1160) and Glu-C (Sigma-Aldrich) [1:100 (w/w), 25 mm ammonium bicarbonate, pH 8.0, overnight at 37 °C]. For Glu-C a 50 mm potassium phosphate solution pH 8.0 was used, as a digestion buffer.

The activity of trypsin, Asp-N and Glu-C was quenched by 1:10 acidification with 10% TFA (Sigma-Aldrich, T6508-500ML). For all enzymes, the resulting peptide mixtures were sequentially extracted from gel, by increasing % w/w of ACN. ACN was removed by vacuum centrifugation for 40 min at 60 °C using a SpeedVac^TM^ Concentrator (Thermo Fisher Scientific, Denmark) and peptide mixtures were loaded on an in-house packed StageTip C_18_ cartridges.

Peptides were eluted in 40% ACN/0.1% FA. The organic phase was removed by vacuum centrifugation for 15 min at 60 °C in a SpeedVac^TM^ Concentrator (Thermo Fisher Scientific, Denmark). Samples were then diluted to approx. 0.1 µg/µL concentration with 0.1% TFA in 5% ACN.

##### Mammoth Bone Fraction in-Solution Digestion

The concentration of each protein fraction (the pellet, the supernatant and pellet+supernatant) was estimated with BCA assay (Thermo Fisher Scientific, 23225). Samples were diluted to approx. 0.3 M GndCl final concentration and 12 µg of protein per fraction were digested with: a) 1:50 w/w ProAlanase prototype for 2 h at pH 1.5, 37 °C; b) 1:100 w/w trypsin (Sigma-Aldrich, T6567) for 18h at pH 8.5, 37 °C under agitation. Tryptic peptides were acidified 1:10 with 10% TFA (Sigma-Aldrich, T6508-500ML). Both ProAlanase and tryptic samples were spun down for 30 min at 17000 × *g* and purified on an in-house packed StageTip C_18_ cartridges. Peptides were purified by three consecutive washes with 0.1% FA, eluted in 40% ACN and evaporated in a SpeedVac^TM^ Concentrator (Thermo Fisher Scientific, Denmark) until ∼ 30 μl final volume. Peptide concentration was determined with a NanoDrop spectrophotometer (Thermo, Wilmington, DE) and samples were diluted to 0.1 µg/µL concentration with 0.1% TFA in 5% ACN.

##### NIST mAb Digestion

Nonreducing proteolytic digestions of NIST mAb (mAb) were performed using either ProAlanase prototype or trypsin. For ProAlanase digestion, 20 µg of NIST mAb were diluted in 0.032 M HCl (pH 1.5) to a final protein concentration of 0.4 mg/ml and ProAlanase was added in a 1:40 (w/w) enzyme-to-protein ratio. The reaction was incubated for 2 h at 37 °C under intense agitation. For tryptic digestion, 20 µg of the antibody were diluted in 10 mm sodium phosphate (NaH_2_PO_4_/Na_2_HPO_4_) buffer (pH 7.4) to a final protein concentration of 0.4 mg/ml. Trypsin was added in a 1:20 (w/w) enzyme-to-protein ratio and the reaction was incubated overnight at 37 °C and quenched by addition of FA to a final concentration of 2% (w/w). ProAlanase digests were quenched by loading peptides directly on a reversed-phase C_18_ LC column.

##### Mass Spectrometry and Liquid Chromatography

All samples, except for the mAb digests, were analyzed on an EASY-nLC^TM^ 1200 system (Proxeon, Odense, Denmark) coupled to a Q Exactive HF-X (34) instrument (Thermo Fisher Scientific, Bremen, Germany), equipped with a nano-electrospray source. Peptides were separated on a 15 cm analytical column (75-μm inner diameter) in-house packed with 1.9 μm C_18_ beads (Reprosil-AQ Pur, Dr. Maisch, r119.b9). The column temperature was maintained at 40 °C, using an integrated column oven (PRSO-V1, Sonation). For optimization, K562 control digestion, multi-enzymatic HeLa analysis and in-solution (IS) *versus* protein aggregation capture (PAC) experiments we used a 33 min gradient at a flow rate of 350 nL/min ramping from 10% buffer B (80% ACN/0.1% formic acid) to 30% B in 25 min, to 45% B in 5 min, to 80% B in 0.5 min and kept 2.5 min. For mammoth bone protein analysis 77 min gradient at a flow rate of 250 nL/min was used. The gradient ramped from 5% B to 30% in 50 min, from 30% to 45 in 10 min, from 45% to 80% in 2 min, kept 5 min, and finally from 80% to 5% in 5 min and kept 5 min. For N3ICD protein analysis we also used a 77-min gradient ramping from 10% B to 30% in 50 min, from 30% to 45% in 10 min, from 45% to 80% in 2 min, kept 5 min, re-equilibrated back to 5% B and kept 5 min respectively. The mass spectrometer was operated in positive ion mode, using data-dependent higher-energy collision induced dissociation (ddHCD) acquisition (35) to automatically isolate and fragment topN multiply charged precursors according to their intensities. Before and after each MS/MS run two successive MS/MS-blank runs were included in the sample queue to prevent carryover contamination between the samples. These consisted of an MS/MS run with an injection exclusively of the buffer used to re-suspend the samples (0.1% TFA and 5% ACN).

The samples for disulfide bond mapping experiment were analyzed using an in-source reduction (ISR) during the electrospray process (LC-ISR-MS/MS) (36, 37). The LC–MS setup was comprised of a Waters Acquity Classic UPLC (Waters Corporation, Wilmslow, UK) connected to an Orbitrap Fusion Lumos (Thermo Fisher Scientific, San Jose, US) MS instrument. The analytical column used was an Acquity UPLC CSH (Waters Corporation, Wilmslow, UK) C_18_ reversed-phase column, 1.7 μm, 1.0 × 150 mm, at the temperature of 55 °C. Peptide separation was conducted using a 77 min gradient at a flow rate of 100 µL/min. The LC gradient started at 4% buffer B (100% ACN/0.1% FA) during the first 4 min followed by linear increase to 55% B at 77 min. The Orbitrap Fusion MS instrument was operated in positive ion mode using H-ESI ion source. The source settings for simultaneous ionization and ISR were: spray voltage of 3.5 kV, sheath, auxiliary and sweep gasses of 15, 10, and 0 arbitrary units respectively, ion transfer tube temperature of 300 °C and vaporizer temperature of 135 °C. The LC-ISR-MS/MS experiments were performed using ddHCD, operated in top speed mode with cycles of 2 s.

##### Data Analysis

All mass spectrometric raw data are available via ProteomeXchange with identifiers PXD019039, PXD021191 and PXD021703.

The raw LC–MS/MS data were processed with MaxQuant (38) v1.6.1.1 (optimization experiments, multi-enzymatic HeLa digests, K562 digests, IS *versus* PAC experiments and Pleistocene mammoth samples) and v1.6.5.0 (N3ICD samples), using the Andromeda search engine. The data generated in optimization, multi-enzymatic HeLa digests, K562 digests and IS *versus* PAC experiments were searched against the reviewed *Homosapiens* UniProt/Swiss-Prot proteome (proteome ID: UP000005640) with 20407 entries (canonical fasta only), downloaded 2018-09-13. Mammoth data were searched against *Loxodonta Africana* UniProt proteome (proteome ID: UP000007646) containing 25894 entries (including protein isoforms), downloaded 2018-09-12. The N3ICD data were searched against *Homosapiens* UniProt/Swiss-Prot proteome (proteome ID: UP000005640) with 20935 entries, including the entries for FLAG tag and a murine intracellular domain of Notch3 receptor (>sp|Q61982|1663-2318), downloaded 2019-04-01.

Depending on digestion conditions, the enzyme was specified as either manually configured “ProAlanase” with cleavage specificity toward the C-term of Pro and Ala, or “Trypsin/P,” “Glu-C,” and “Asp-N.” Tryptic searches included maximum two missed cleavages, whereas for ProAlanase, Glu-C and Asp-N human data analysis missed cleavage was increased to four, and for ProAlanase Pleistocene mammoth bone and N3ICD data – to ten (supplemental Fig. S1).

**Fig. 1.**
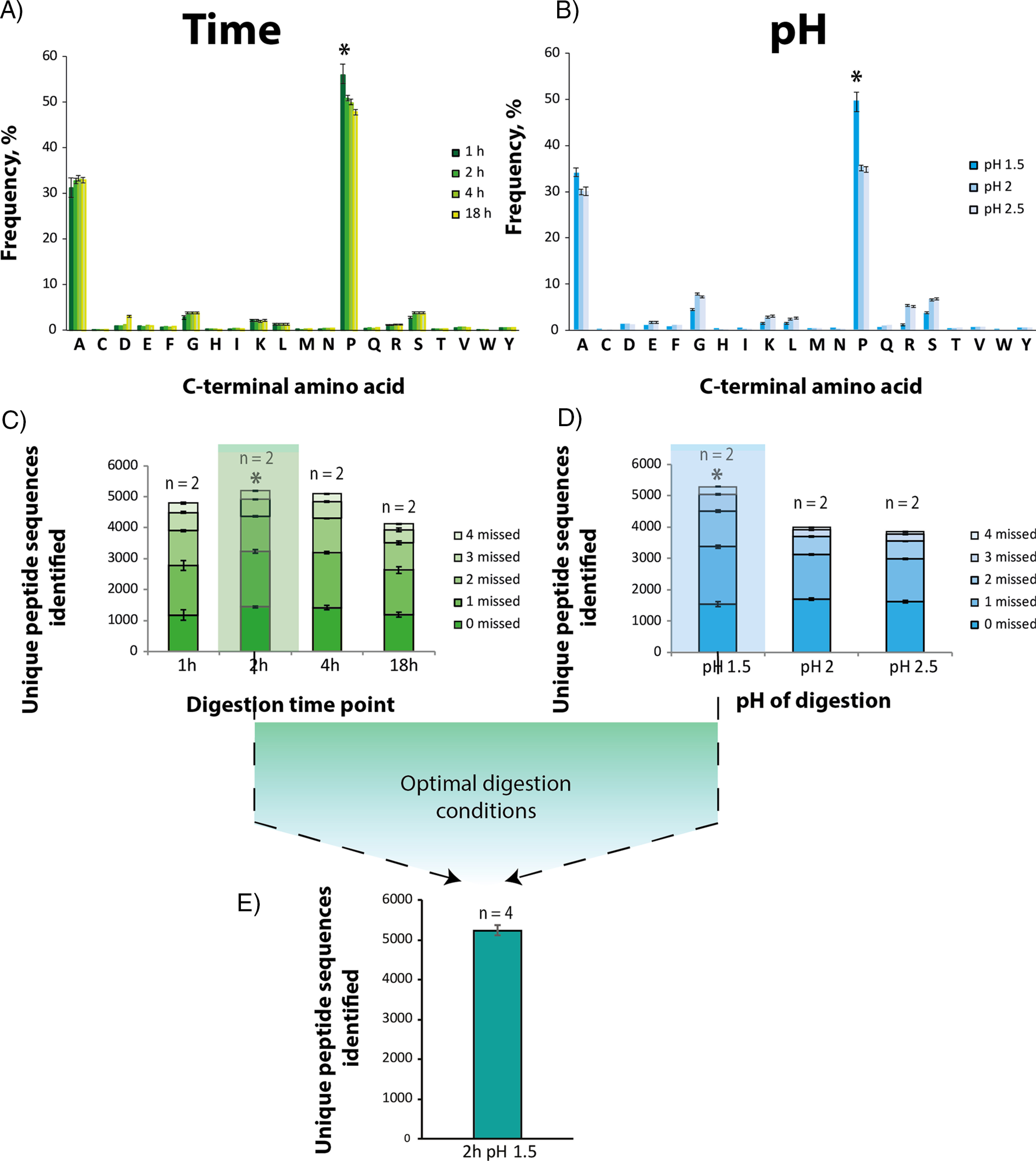
**Determination of optimal digestion conditions for ProAlanase, using HeLa cell lysate.** Specificity plots reflecting the average C-terminal amino acid frequency in ProAlanase-digested HeLa cell lysates (*n* = 2) at the different pH (*B*) and time (*A*) of digestion. Unspecific MaxQuant search settings were used for specificity estimation. C-terminal amino acid frequency on the *y* axis was calculated as a relative count of identified peptides, containing the specific C-terminal residue. Average proportion of unique peptides identified in ProAlanase digests of HeLa cell lysates (*n* = 2) carrying 0, 1, 2, 3 or 4 missed cleavage sites, using enzyme-specific settings for peptide-to-sequence matching. The comparison is done for the different pH (*D*) and time (*C*) of digestion. Digestion conditions, considered as optimal, are marked with the *. Unique peptides sequences identi_ed in ProAlanase digests of HeLa cell lysates (*n* = 4) at the optimal digestion conditions (*E*).

To assess the specificity of ProAlanase, an “unspecific” MaxQuant search was conducted.

Carbamidomethylation of cysteine was specified as fixed modification for all searches. For human data analysis such variable modifications, as oxidation of methionine, protein N-terminal acetylation, deamidation of asparagine and glutamine and glutamine-to-pyroglutamate conversion were considered. To map histone-specific PTMs in a second pass database search, arginine methylation, phosphorylation of serine/threonine/tyrosine sites, and ubiquitylation, acetylation, mono-, di- and tri-methylation of lysines were specified as protein specific variable modifications for the histone sequences identified in the initial MaxQuant searches.

For mammoth data search methionine oxidation, deamidation of asparagine and glutamine, proline hydroxylation and glutamine-to-pyroglutamate conversion were specified as variable modifications.

For N3ICD data analysis protein N-terminal acetylation, oxidation of methionine, phosphorylation of serine, threonine, and tyrosine, as well as deamidation of asparagine and glutamine residues, were set as variable modifications.

Maximum number of modifications was set to 5 for all searches. Mass tolerance of 20 parts per million (ppm) or 0.05 daltons (Da) was set for the first search of precursor ions, followed by 4.5 ppm for main search after mass re-calibration. Twenty ppm mass tolerance was set for fragment ions. A minimum peptide length of 7 amino acids was required for all identifications. A minimum Andromeda score was set to 20 for all optimization experiments, whereas for the Pleistocene mammoth bone analysis it was 40, both for unmodified and modified peptides. For N3ICD protein analysis the Andromeda score was 25 for unmodified and modified peptides. Pleistocene mammoth bone protein matches were accepted with a minimum of two unique peptide sequences identified.

False discovery rate (FDR) was set to 1% on peptide spectrum match (PSM), PTM site and protein level. To determine and control the number of false-positive identifications, MaxQuant applies a target-decoy search strategy, using the concept of posterior error probability (PEP) to integrate multiple peptide properties, such as length, charge, number of modifications, and Andromeda score into a single quantity reflecting the quality of a PSM (38, 39).

The raw LC–MS/MS files from disulfide bond mapping experiment were analyzed using Byonic (40) (Protein Metrics, San Carlos, CA) software. We used a simple fasta file containing only the light and the heavy chains of NIST mAb for searching the data. For ProAlanase data analysis a completely unbiased, unspecific search was used as a starting point. The second iteration included semi-specific search, considering cleavage after proline, alanine, serine, and glycine residues. The number of maximum missed cleavages was unlimited, but the maximum precursor mass was set to 10 kDa. The tryptic data were only searched with unspecific digestion settings. No FDR cutoffs were used, as above mentioned data searches were primarily used for identification of peptide constituents of disulfide-containing chromatographic peaks. Using the chromatogram as a starting point, all major peaks should be explainable, especially when performing an in-depth protein characterization in biopharmaceutical industry.

*De novo* sequencing of the N3ICD protein was performed using PEAKS X+ Studio (41). Enzyme specificity was set to either trypsin, Glu-C and Asp-N or ProAlanase and maximum missed cleavage was set to 2, 4, 4, and 10, respectively. Carbamidomethylation of cysteine was specified as fixed modification, whereas protein N-terminal acetylation, oxidation of methionine, phosphorylation of serine, threonine, and tyrosine and deamidation of asparagine and glutamine residues, were set as variable modifications. The number of max variable PTMs per peptide was set to 5. Precursor mass error tolerance was set to 10.0 ppm and fragment mass error tolerance to 0.02 Da. After the search was completed, the multi-enzymatic *de novo* peptides (“*de novo* peptides.csv”) were filtered using *De novo* Score ≥ 90%, removing duplicate entries, merged in all possible combinatorial combinations and imported into PepExplorer (42) within PatternLab (43) for Proteomics v4.1.1.21 platform for further re-scoring and alignment to the reference protein sequences. For this, a reference *Homosapiens* UniProt/Swiss-Prot proteome (proteome ID: UP000005640) with 20935 entries, including a murine intracellular domain of Notch3 receptor and common contaminants, was used.

##### Experimental Design and Statistical Rationale

The number of experimental (process) replicates and biological replicates were specified, as “*n = x*”, for all the experiments conducted, and reflected in corresponding figures. “Figure Legends” contain a more detailed description of statistical tests and criteria for determining significance in each specific case.

Model experiments, conducted for determination of optimal digestion conditions and specificity of ProAlanase, were performed in experimental duplicates with seven different conditions (Fig. 1), resulting in analysis of 14 samples. The same biological sample (HeLa protein extract) was used in all optimization experiments to limit the variation to sample preparation procedure only. Human protein extract was equally aliquoted 14 times for duplicate analysis of different digestion conditions, leading to 14 samples. For the optimal digestion conditions (2 h at pH 1.5) four experimental replicates (Fig. 1*E*) were processed, using the same biological sample.

A control K562 cell lysate digestion was performed in experimental triplicates at the optimal digestion conditions, resulting in analysis of 3 samples (supplemental Fig. S2). The same biological sample (K562 protein extract) was equally aliquoted 3 times and processed in parallel.

**Fig. 2.**
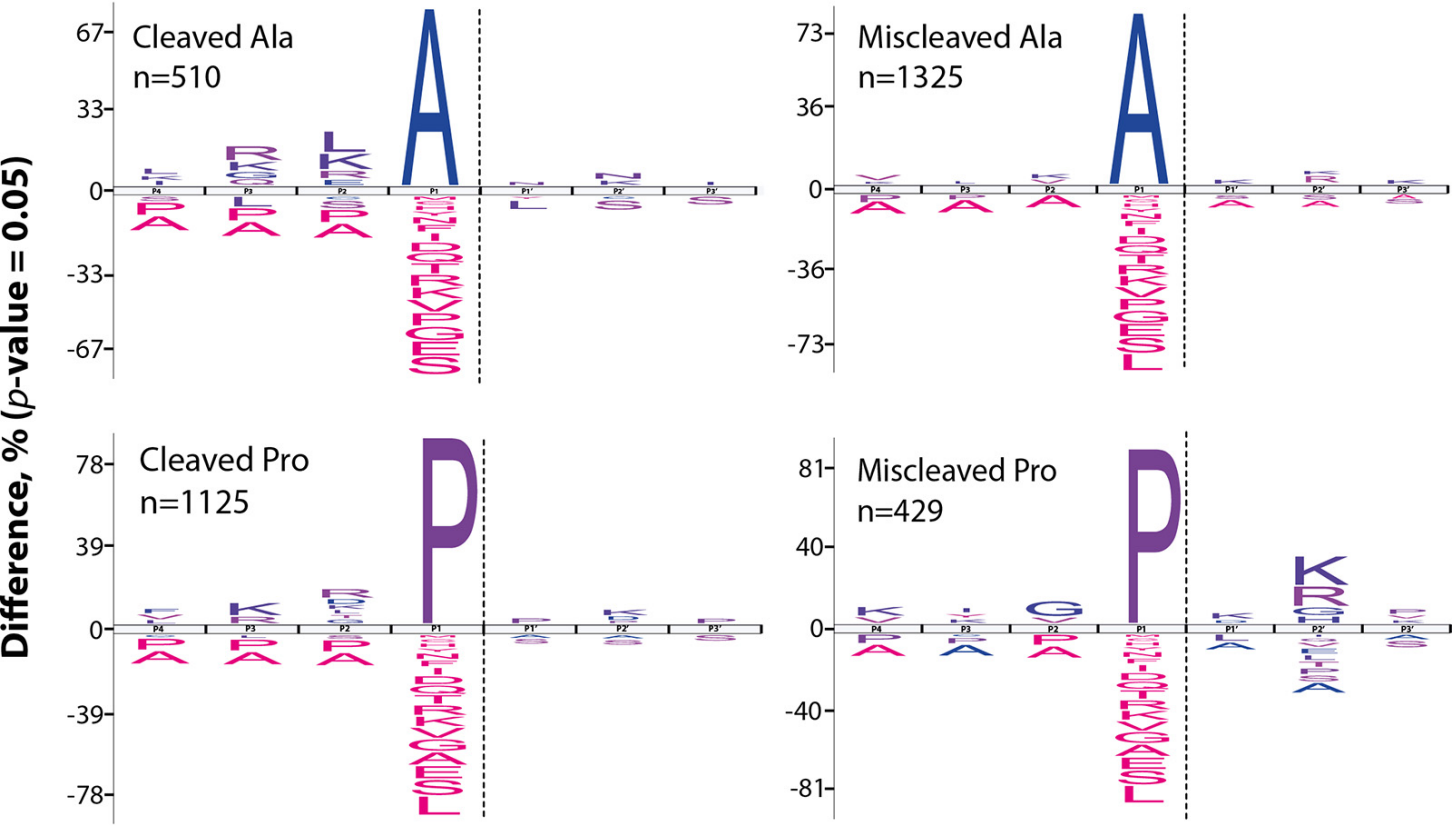
**Characterization of missed cleavage patterns in ProAlanase-digested HeLa cell lysate.** Frequency distribution plots of cleavage sites identified in human proteome samples, digested with ProAlanase. The unique peptide sequences from the two experimental replicates were concatenated to evaluate sequence context of missed proline and alanine cleavages. Sequences were aligned at cleavage sites between P1 and P1'. The significant differences in amino acid occurrence are compared with the natural abundance in human proteome (*P* < 0.05).

For multi-enzymatic profiling of human proteome, HeLa cell lysate was equally aliquoted 10 times and digested with trypsin in experimental quadruplicates and with rAsp-N and Glu-C - in experimental triplicates, resulting in analysis of 10 samples. For comparison between in-solution and PAC digestion protocols, the same biological sample (HeLa protein extract) was equally aliquoted five times, resulting in analysis of the two experimental replicates of in-solution digestion and three experimental replicates of PAC digestion (supplemental Fig. S9).

Pleistocene mammoth bone digestion was conducted with two different enzymes (ProAlanase and trypsin) in parallel on three separate protein fractions (the pellet, the supernatant and pellet+supernatant), in duplicates. This resulted in analysis of 12 samples (Fig. 4). The same biological sample (Pleistocene mammoth bone powder) was used in all experiments to limit the variation to sample preparation procedure only. Mammoth bone protein extract was equally aliquoted 4 times and each aliquot was manually fractionated into 3 fractions for further duplicate analysis, leading to 12 samples in total. One negative extraction blank was processed alongside with the mammoth bone samples, using each enzyme, in duplicates.

An in-gel digestion of the FLAG immunoprecipitated N3ICD protein band was performed in three biological replicates, digested with ProAlanase and two biological replicates, digested with trypsin, Glu-C and rAsp-N, resulting in analysis of a total of nine samples (Fig. 5*B*).

For disulfide bond mapping 3 experimental replicates of mAb digestion either with ProAlanase or with trypsin were used. Same biological sample (mAb) was used to limit the variation to sample preparation procedure only. mAb protein extract was equally aliquoted 6 times for duplicate analysis of two enzymatic digests, leading to 6 samples in total.

## RESULTS

### 

#### 

##### Optimization Experiments Allow to Determine Optimal Digestion Conditions for ProAlanase

To determine the best digestion conditions for the ProAlanase, we conducted a series of optimization experiments for in-solution digestion. We tested various solution pH (1.5, 2.0 and 2.5) and time points of digestion (1, 2, 4 and 18 h). All optimization experiments were performed on HeLa cell lysate digested with ProAlanase, as described in the methods section. The purified peptides were analyzed by nanoLC-MS/MS and the raw MS data were processed using the MaxQuant software with both ProAlanase-specific and unspecific peptide search settings. The unspecific search was used to assess the specificity of the enzyme under different digestion conditions (Fig. 1*A*,^1^*B*), whereas a specific search was used to compare the number of total peptide sequences identified (Fig. 1*C*, 1*D*). The results showed that the enzyme cleaves primarily after alanine (∼30% frequency) and proline (∼50% frequency). The highest specificity toward both amino acids was observed at pH 1.5 1 h of digestion, whereas the highest numbers of unique peptides were identified at pH 1.5 2 h digestion. Based on this, pH 1.5 2 h digestion was considered as optimal (Fig. 1*E*). Importantly, specificity for C-terminal proline decreased from 49 to 35% and for C-terminal alanine from 34 to 30% as digestion pH increased from 1.5 to 2.5 (Fig. 1*B*). This points to a dramatic decrease in cleavage specificity as the pH of digestion mixture is increased by 0.5 units. Furthermore, we performed a control digestion of K562 cell lysate at the optimal digestion conditions at pH 1.5 for 2h using urea as a lysis buffer, instead of GndCl. The results showed comparable peptide identifications, cleavage specificity and reproducibility to the ones observed with our standard GndCl workflow (supplemental Fig. S2).

Next, we evaluated the sequence context of missed Pro and Ala cleavages. The obtained sequence motif plots using iceLogo (44) reflected inverse miscleavage patterns for Pro- and Ala-peptides, generated at optimal digestion conditions. The number of miscleaved alanines was ∼2 times higher than the number of cleaved Ala. In contrast, twice as many proline residues were cleaved compared with the miscleaved ones (Fig. 2). Interestingly, we observed an over-representation of basophilic amino acid residues in P + 2 position for missed-cleaved proline residues. Conversely, a similar overrepresentation of basophilic amino acid residues in P-2 position appear to promote cleavage after alanine. This mirrored cleavage specificity pattern suggests a fundamental difference in the recognition of the two substrate amino acids by the protease.

Tryptic data were used, as a reference (supplemental Fig. S3), and reflected the expected miscleavage patterns (45).

**Fig. 3.**
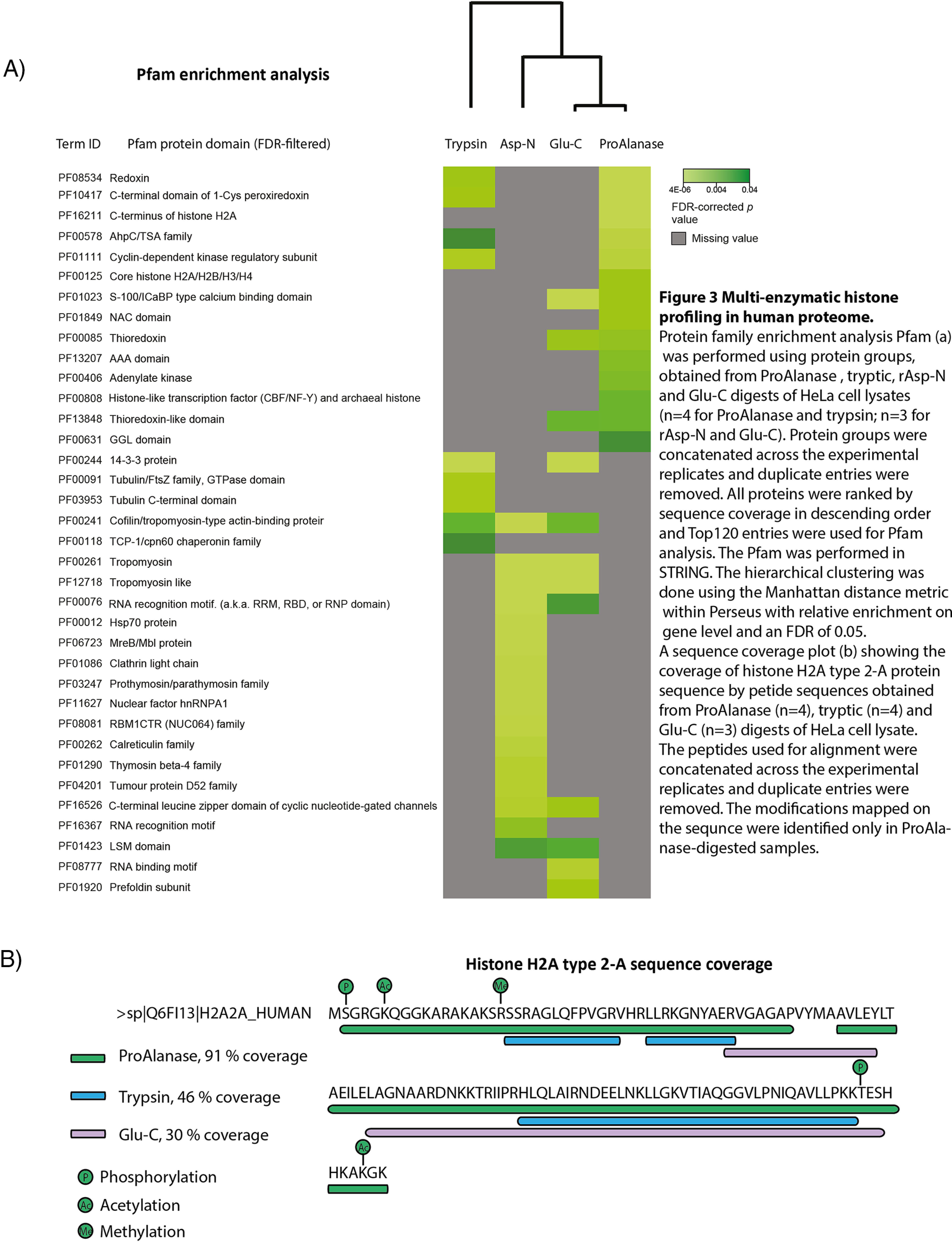
**Multi-enzymatic histone profiling in human proteome.** Protein family enrichment analysis Pfam (*A*) was performed using protein groups, obtained from ProAlanase, tryptic, rAsp-N and Glu-C digests of HeLa cell lysates (*n* = 4 for ProAlanase and trypsin; *n* = 3 for rAsp-N and Glu-C). Protein groups were concatenated across the experimental replicates and duplicate entries were removed. All proteins were ranked by sequence coverage in descending order and Top120 entries were used for Pfam analysis. The Pfam was performed in STRING. The hierarchical clustering was done using the Manhattan distance metric within Perseus with relative enrichment on gene level and an FDR of 0.05. A sequence coverage plot (*B*) showing the coverage of histone H2A type 2-A protein sequence by petide sequences obtained from ProAlanase (*n* = 4), tryptic (*n* = 4) and Glu-C (*n* = 3) digests of HeLa cell lysate. The peptides used for alignment were concatenated across the experimental replicates and duplicate entries were removed. The medications mapped on the sequence were identified only in ProAlanase-digested samples.

##### ProAlanase Improves Histone Sequence Coverage and Their PTMs

To assess the proteome-wide use of ProAlanase, we performed Protein Family (Pfam) enrichment analysis (46) on the high-sequence coverage proteins identified from the HeLa cell lysates digested with multiple proteases. The results showed a high complementarity of ProAlanase-covered protein domains, compared with tryptic, Asp-N and Glu-C. Importantly, several histone domains (C terminus of histone H2A, Core histone H2A/H2B/H3/H4 and Histone-like transcription factor (CBF/NF-Y) and archaeal histone) were enriched exclusively in ProAlanase-digested HeLa cell lysate (Fig. 3*A*).

Furthermore, we generally observe a higher sequence coverage over a broader histone sub-family member range for ProAlanase-digested samples, compared with trypsin, Glu-C and Asp-N (supplemental Table S1). In particular, ProAlanase appears to be exceptionally suitable for the analysis of histone H2A family members, enabling sequence coverage up to 91%, which is at least twice as much as for any of the other proteases (Fig. 3*B*). Moreover, the increase in the H2A sequence coverage by ProAlanase also allowed for identification of several histone specific PTMs, including the known (11) lysine acetylation, arginine methylation, serine and threonine phosphorylation sites, which none of the other protease digestion samples were able to identify.

##### ProAlanase Improves Identification of NonCollagenous Proteins in Pleistocene Mammoth Bone

Type I collagen is the most abundant and well-studied collagen. It constitutes more than 90% of bone organic mass and is the major protein of tendons, skin, ligaments, cornea, and many interstitial connective tissues, except for hyaline cartilage, brain, and vitreous body (47, 48).

Although collagen preserves better than the other bone proteins and is often detected in archaeological bone (15), its value as a phylogenetic marker is limited because of its low sequence variability across species (49). Accordingly, it masks taxonomically more informative noncollagenous proteins and complicates their mass spectrometric detection (15), ultimately leading to a significant loss of phylogenetic signal. For this reason, workflows for efficient collagen depletion and improved noncollagenous bone protein identification are highly sought after.

Here, we hypothesized that by using ProAlanase collagen could be efficiently digested into shorter amino acid entities while at the same time improving identification of noncollagenous bone proteins. To test this hypothesis, we applied the optimized ProAlanase digestion workflow to previously characterized Pleistocene mammoth bone sample (50). In brief, the experiment was performed on a demineralized bone sample, extracted in 6M GndCL and subject to manual fractionation to access both soluble and insoluble proteins. Each fraction was digested either with ProAlanase or with trypsin as a reference. The resulting peptides were analyzed by nanoLC-MS/MS and the obtained raw MS data were processed with the MaxQuant software.

**Fig. 4.**
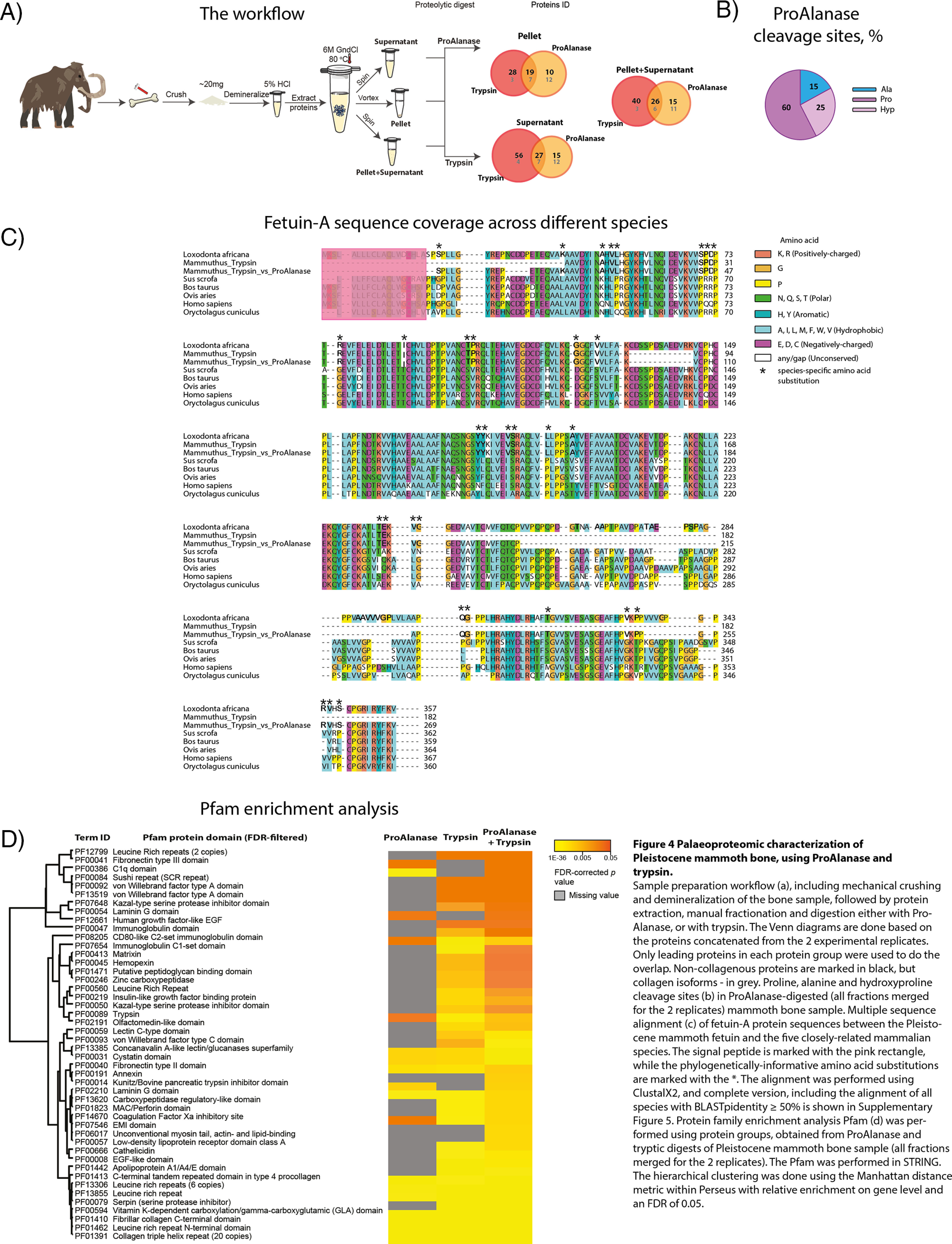
**Palaeoproteomic characterization of Pleistocene mammoth bone, using ProAlanase and trypsin.** Sample preparation workflow (*A*), including mechanical crushing and demineralization of the bone sample, followed by protein extraction, manual fractionation and digestion either with Pro- Alanase, or with trypsin. The Venn diagrams are done based on the proteins concatenated from the 2 experimental replicates. Only leading proteins in each protein group were used to do the overlap. Noncollagenous proteins are marked in black, but collagen isoforms - in gray. Proline, alanine and hydroxyproline cleavage sites (*B*) in ProAlanase-digested (all fractions merged for the 2 replicates) mammoth bone sample. Multiple sequence alignment (*C*) of fetuin-A protein sequences between the Pleistocene mammoth fetuin and the five closely related mammalian species. The signal peptide is marked with the pink rectangle, whereas the phylogenetically-informative amino acid substitutions are marked with the *. The alignment was performed using ClustalX2, and complete version, including the alignment of all species with BLASTpidentity ≥ 50% is shown in supplemental Fig. S5. Protein family enrichment analysis Pfam (*D*) was performed using protein groups, obtained from ProAlanase and tryptic digests of Pleistocene mammoth bone sample (all fractions merged for the 2 replicates). The Pfam was performed in STRING. The hierarchical clustering was done using the Manhattan distance metric within Perseus with relative enrichment on gene level and an FDR of 0.05.

Overall, the results showed similar protein distribution across the different fractions of mammoth bone extract, both for ProAlanase and tryptic digests (supplemental Fig. S4*A*). However, comparing proteins identified in each fraction (pellet, supernatant and pellet+supernatant) between the two different proteases, we observed both complementary noncollagenous proteins and complementary collagen isoforms (Fig. 4*A*). These results supported our initial hypothesis, as well as revealed the ability of ProAlanase to cleave not only proline, but also hydroxyproline sites (Fig. 4*B*) highly prevalent in collagen (16, 51). Next, we merged the raw MS files in a single MaxQuant search - by protease and in combination, to increase the coverage of identified proteins. The results showed an expected increase in protein sequence coverage, when combining tryptic and ProAlanase data, compared with each of the proteases alone (supplemental Fig. S4*B*). Most importantly, combining the data generated by two enzymes allowed to increase the sequence coverage for proteins containing species-specific amino acid substitutions (supplemental Table S2), which is crucial for reliable ancient species identification and their correct phylogenetic placement. Fig. 4, c demonstrates an alignment of multiple fetuin-A sequences (MSA (52)), corresponding to a partially-reconstructed Pleistocene mammoth fetuin and the reference sequences from five other related mammalian species. The MSA shows that using ProAlanase in combination with trypsin allows to cover almost twice as many variable regions in Pleistocene mammoth fetuin-A, compared with tryptic data only.

To compare protein domains covered by two enzymes, separately and in combination, the merged data sets were also subject to Pfam enrichment analysis. The results revealed that several complementary noncollagenous protein domains (Kunitz/Bovine pancreatic trypsin inhibitor domain, Fibronectin type III, C1q and Laminin G domain) for ProAlanase and tryptic samples (Fig. 4*D*), most likely because different protein parts were covered by each protease. Surprisingly, the merged “ProAlanase+trypsin” data returned both “tryptic” and “ProAlanase” domains, as well as several additional examples, not covered neither by ProAlanase, nor by trypsin (Annexin, EMI and Unconventional myosin tail, actin- and lipid-binding domain) (Fig. 4*D*). The different protein domains identified can be useful for better understanding of biological processes in ancient specimens.

Similarly to the human proteome analysis, ProAlanase showed complementarity to trypsin in analysis of the Pleistocene mammoth bone proteome demonstrating the potential of this protease for palaeoproteomics applications.

##### ProAlanase Improves Sequence Coverage and Phosphorylation Site Profiling in Proline-Rich Protein

Because most proteins are phosphorylated, many on multiple sites (53), the characterization of phosphorylation has become a paramount issue for understanding the fine-tuning of protein expression and activity. One notable example is represented by the Notch receptor's family (54).

Four Notch paralogs (Notch1-4) are encoded in mammalian genomes. They are single-pass transmembrane proteins that share a characteristic three-domain structure: an extracellular domain (NECD), a transmembrane region (NTM) and an intracellular domain (NICD), which is able to translocate into the nucleus and activate transcription of target genes (55). NICD activity is fine-tuned (56), for instance by dynamic PTMs (54). However, little is known about the phosphorylation of the intracellular domain of Notch3 paralog.

To comprehensively map phosphorylation sites and to determine their stoichiometry in a purified protein by LC–MS/MS, it is essential to obtain its full sequence coverage (57). An *in silico* digestion of Notch3 intracellular domain (N3ICD) protein resulted in a number of very long tryptic polypeptides, which are likely to be difficult to analyze by LC–MS/MS. Conversely, N3ICD *in silico* digestion with ProAlanase produced shorter peptides, potentially more amenable for LC–MS/MS analysis (supplemental Fig. S6). This discrepancy between trypsin and ProAlanase *in silico* digests can be explained by a lower frequency of arginines and lysines, but high representation of proline cleavage sites in the N3ICD protein sequence.

We aimed at reaching complete coverage of N3ICD sequence for efficient phosphorylation profiling in this protein. To achieve this, we performed an over-expression of a FLAG-tagged version of N3ICD in HEK293T cells. After immunoprecipitation, N3ICD and the background proteins were in-gel digested either with ProAlanase or with trypsin as a reference. Desalted and concentrated peptides were analyzed by LC–MS/MS and the raw MS data were processed using the MaxQuant software.

**Fig. 5.**
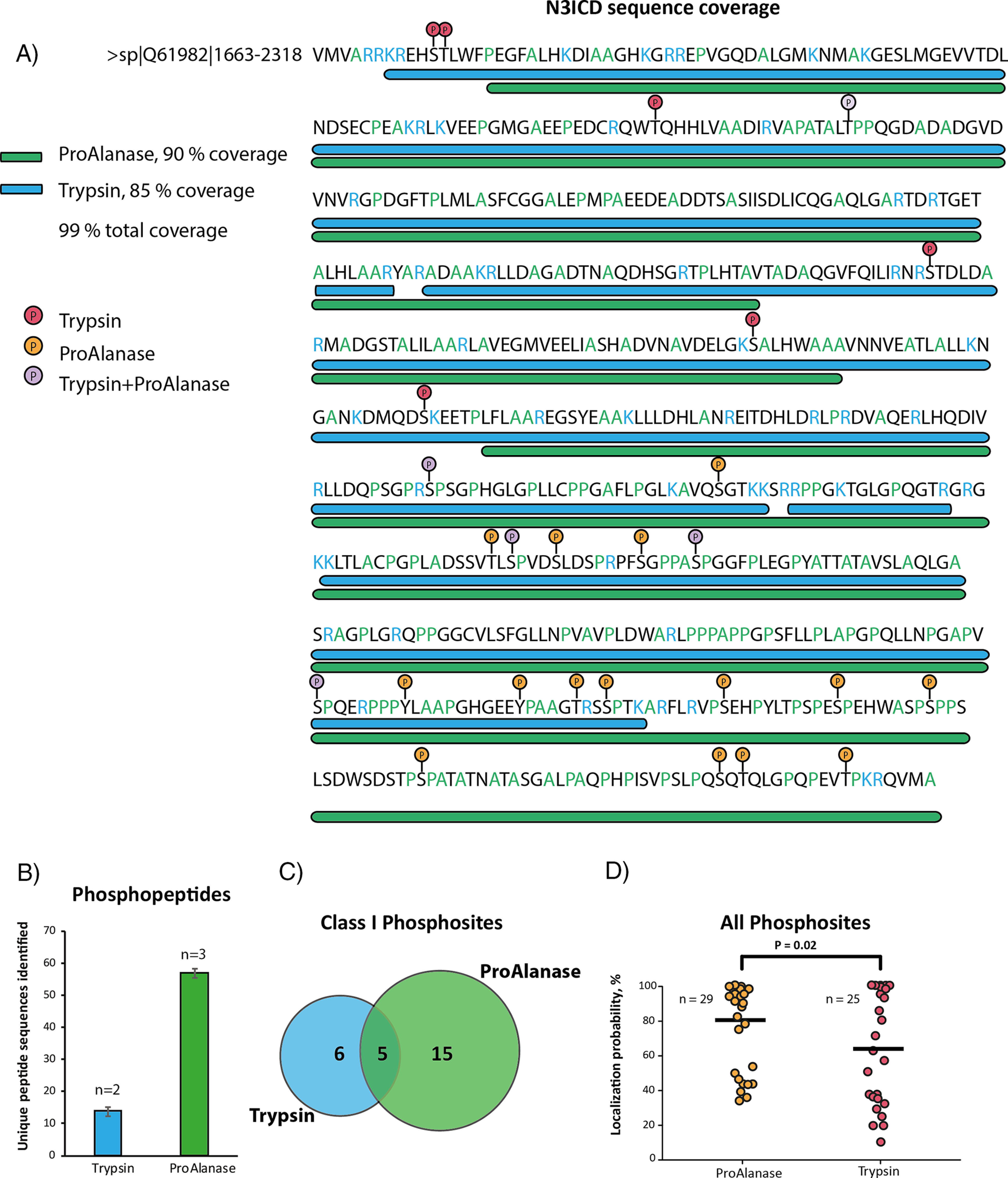
**Phosphorylation profiling in immunoprecipitated N3ICD protein.** A sequence coverage plot (*A*) showing the coverage of N3ICD protein sequence by concatenated ProAlanase and tryptic peptides obtained from in-gel digestion of N3ICD protein immunoprecipitates (*n* = 2). Average number of unique phosphopeptide sequences (*B*) identified in ProAlanase (*n* = 3) and tryptic (*n* = 2) digests of N3ICD immunoprecipitates. A Venn Diagram (*C*) showing an overlap of class I (localization probability ≥ 75%) phosphorylation sites in ProAlanase- and trypsin digested samples of N3ICD protein immunoprecipitates (*n* = 2). A scatter plot (*D*) of all phosphorylation sites identi_ed in ProAlanase and tryptic digests of N3ICD protein (*n* = 2). The variances were estimated using the two-sample Fisher's exact test and the significance was determined with the one-tailed two-sample *t* test (α = 0.05) with equal variance. The mean value is highlighted as a dash and n corresponds to the number of unique phosphorylation sites identified with each of the proteases.

The mass spectrometric analysis of the ProAlanase digestion mixture resulted in 90% sequence coverage of the N3CID protein, whereas tryptic peptides covered 85% of the sequence (Fig. 5*A*). The reason why none of the proteases allowed for a 100% coverage is likely explained by the fact that some of N3ICD regions contain fewer KR or AP residues, than the rest of the sequence. For example, the C terminus of N3ICD protein contains multiple A and P cleavage sites and almost no K and R residues (Fig. 5*A*) resulting in complete C-terminal coverage by ProAlanase and no tryptic coverage, respectively. Notably, merging tryptic and ProAlanase raw MS files in a single MaxQuant search increased total protein sequence coverage to 99% (Fig. 5*A*). This essentially complete sequence coverage, obtained by combining ProAlanase and tryptic peptides, confidently mapped 26 phosphorylation sites in the N3CID protein without prior phosphopeptide enrichment. Importantly, four times more unique phosphopeptides (Fig. 5*B*) and twice as many class I localized phosphosites (Fig. 5*C*) were identified in ProAlanase digests, compared with trypsin. We also observed on average a higher quality of phosphosite localization using ProAlanase, compared with trypsin. This conclusion was derived from a significantly higher mean value of localization probability (Fig. 5*D*), calculated using all phosphorylation sites identified in ProAlanase and tryptic digests of N3ICD protein.

This example demonstrates a high potential of ProAlanase in phosphorylation profiling in a proline-rich affinity purified exogenously expressed protein.

##### ProAlanase Digestion at Low-pH Enables Accurate Disulfide Bond Mapping of a Monoclonal Antibody

Because ProAlanase is active at the pH 1.5, this protease should be, potentially, suitable for accurate disulfide bond mapping. The low pH optimum and short incubation time of the protease could be highly beneficial for minimizing or even avoiding disulfide scrambling during the digestion procedure (58). To assess the suitability of ProAlanase for disulfide bond mapping purposes, a nonreducing proteolytic digestion of the NIST mAb (mAb) was performed. The antibody was digested with ProAlanase, using the optimal digestion conditions, and with trypsin as a reference. Next, the samples were analyzed by online liquid chromatography in-source reduction tandem MS (LC-ISR-MS/MS), using previously described strategies for data acquisition (36) and analysis (37), to identify the reduced cysteine-containing peptides and disulfide-bonded species.

**Fig. 6.**
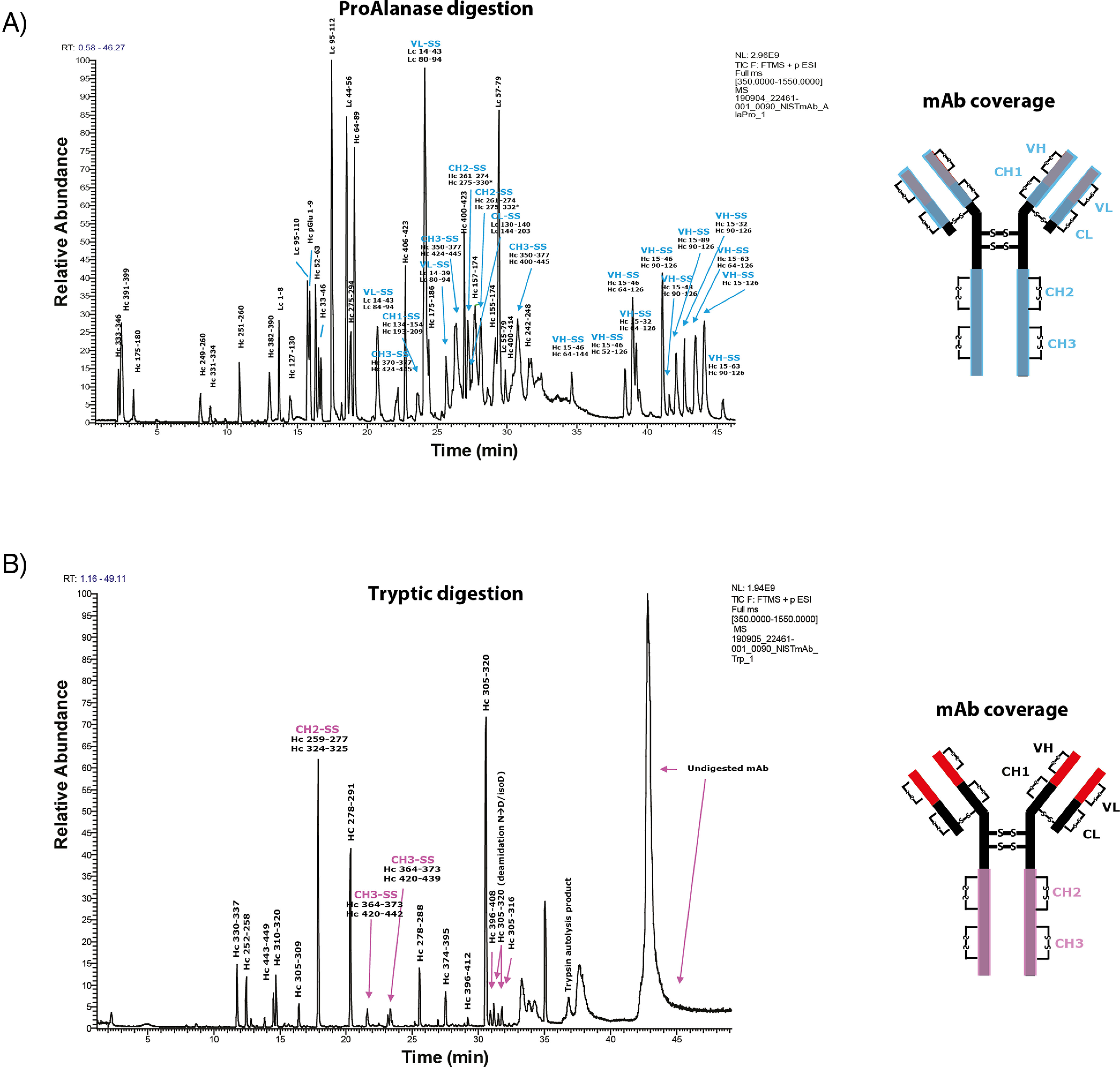
**Disulfide bond mapping in NIST mAb, using ProAlanase and trypsin.** Total ion current (TIC) chromatograms of ProAlanase-and trypsin-digested mAb. Both constant and variable regions (except for the hinge region) are covered by ProAlanase peptides (*A*); only constant region is covered by the tryptic peptides (*B*).

The resulting chromatograms of ProAlanase and tryptic digestions are shown in Fig. 6*A*, 6*B*, respectively. One of the key features observed was a higher digestion efficiency of the nonreduced NIST mAb, using ProAlanase despite the use of less enzyme and roughly 8-fold shorter digestion time, compared with trypsin. Highly-abundant peptide peaks derived from entire mAb molecule sequence are reflected in the chromatogram of ProAlanase-digested mAb (Fig. 6*A*), whereas only a few abundant peptide peaks, covering the heavy chain Fc region, are observed in the tryptic chromatogram (Fig. 6*B*). This is likely explained by the fact that tryptic digestion was performed in denaturation-free conditions, whereas the low-pH conditions used for ProAlanase digestion promoted denaturation of the antibody resulting in higher digestion efficiency.

Most importantly, ProAlanase digestion allowed confident identification and assignment of all disulfide bonds in NIST mAb, except for those in the hinge region containing the two heavy chains-linking disulfide bonds and the disulfide bond linking the light chains to the heavy chains. Conversely, only a few of the disulfide bonds were detected in the tryptic digest. An example of a confidently-identified CH1-SS disulfide bond, using ProAlanase digestion, is demonstrated in supplemental Fig. S7, summarizing the spectral information from ISR-induced partial disulfide bond reduction on MS1 level and characterizing the two constituting reduced peptides on MS2 level. The ability of ProAlanase to efficiently digest nonreduced proteins at low pH and short incubation times dramatically limits the risk of introducing artificial disulfide scrambling during the sample preparation and allows for efficient protein digestion using a less time- and cost-consuming sample preparation procedure.

**Fig. 7.**
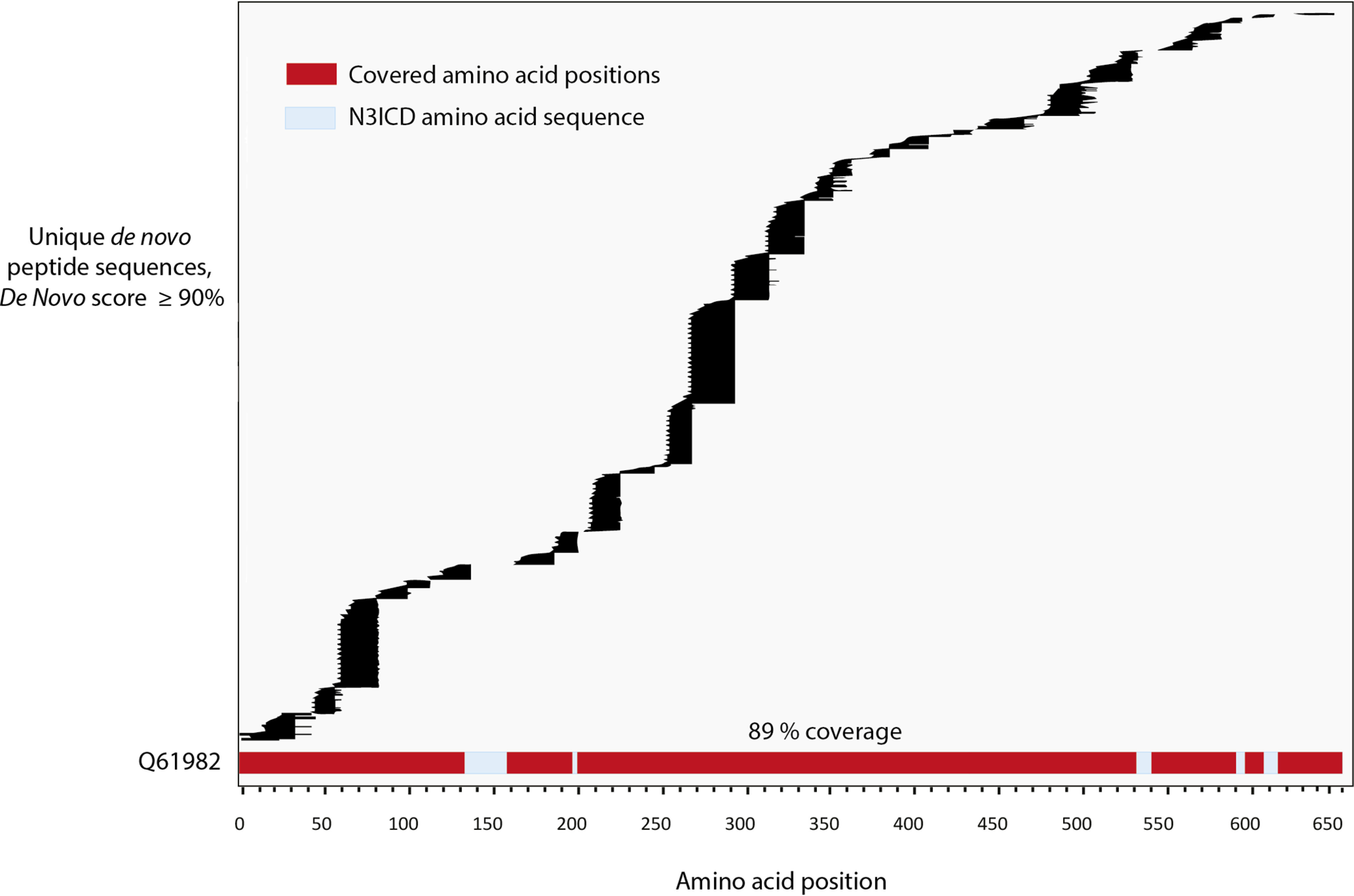
**Database-independent sequence reconstruction of N3ICD protein (Q61982), using ProAlanase and trypsin.** *De novo* sequencing plot showing a 89% coverage of N3ICD protein (Q61982) by unique ProAlanase and tryptic *de novo* peptides, obtained from in-gel digested N3ICD immunoprecipitates. The two biological replicates of ProAlanase- and trypsin-digested N3ICD were merged in as single data search, by protease. PEAKS X+ Studio was used to generate ProAlanase and tryptic *de novo* peptide sequences. The generated peptides were _filtered using a 90% De Novo score threshold and duplicates were removed. Sequence alignment was performed using PepExplorer within PatternLab for Proteomics.

Taken together, this presents ProAlanase as a powerful tool for efficient and accurate disulfide bond mapping of therapeutic proteins.

##### ProAlanase Facilitates a Nearly Complete De Novo Assembly of Exogenously Expressed Purified Protein Sequence

As mentioned previously, to perform an efficient *de novo* sequencing a multi-enzymatic digestion is typically required to produce overlapping peptide sequences (27). Here we hypothesized that using ProAlanase and tryptic digestion could provide sufficient orthogonality in the resulting peptides to enable a full-length *de novo* sequencing of an exogenously expressed affinity purified protein.

To test this hypothesis, we used the previously obtained data set from ProAlanase and trypsin-digested N3ICD protein. We also used GluC- and AspN-generated N3ICD data sets as a reference. In brief, *de novo* sequencing of the N3ICD protein was performed using PEAKS X+ Studio and the obtained high-score multi-enzymatic *de novo* peptides were merged in various combinatorial combinations and imported into PepExplorer for further re-scoring and alignment. The results of *de novo* peptide alignment returned 89% sequence coverage of the N3ICD protein (Fig. 7), using ProAlanase and tryptic *de novo* peptides. When combining *de novo* N3ICD peptides from other proteases 1 + 1 (ProAlanase + Asp-N, trypsin + Asp-N, trypsin + Glu-C, ProAlanase + Glu-C and Glu-C+ Asp-N) a sequence coverage **≤** 80% was observed (supplemental Table S3). A comparable coverage of 89 and 90% was achieved only, when merging *de novo* peptide sequences generated by 3 or 4 proteases (trypsin + ProAlanase + Glu-C, trypsin + ProAlanase + Asp-N and trypsin + ProAlanase + Glu-C + Asp-N, supplemental Table S3). Notably, the intracellular domain of Notch3 protein is comprised of 656 amino acid residues, and 584 of them were confidently covered using *de novo* peptide sequences, produced by ProAlanase and trypsin. To our knowledge, this is the first example of nearly complete *de novo* coverage of a purified exogenously expressed protein, using only two enzymes for proteolytic digestion.

## DISCUSSION

Here we have characterized a proteomics-grade proline- and alanine-specific protease. As shown by van der Laarse *et al.* (30), ProAlanase (also known as An-PEP or EndoPro) is active in a broad pH range from 2 to 5.5, generating various peptide populations at these two pH maxima. Here we show that lowering the pH of the digestion dramatically increases cleavage specificity (Fig. 1*B*). In our hands, the highest digestion specificity and efficiency as well as the highest peptide identifications were observed at the pH of 1.5 in 2h of protein digestion. Under the optimized digestion conditions, ProAlanase displays C-terminal cleavage specificity of approximately 50% for proline and 30% for alanine.

Based on the optimal digestion conditions determined for ProAlanase, we predicted several beneficial application areas for this protease, such as digestion of proline-rich proteins, PTM analysis, disulfide bond mapping, *de novo* protein sequencing and proteomic analysis of histone variants.

It has been shown previously that ProAlanase is useful for accurate phosphorylation profiling in combination with enrichment of phosphorylated peptides before LC–MS/MS analysis (30). Here, we extended the use of ProAlanase for phosphorylation site analysis without the need for phosphopeptide enrichment. ProAlanase digestion showed an improved phosphorylation profiling in proline-rich single protein N3ICD, compared with trypsin, as well as allowed to increase total sequence coverage of the protein by combining peptides generated by both proteases. A similar increase in total protein sequence coverage was observed, when combining the data from ProAlanase and tryptic digests of the Pleistocene mammoth bone sample. Importantly, this allowed to increase the sequence coverage for noncollagenous proteins containing species-specific amino acid substitutions relevant for phylogenetic placement. ProAlanase also appears to be highly suitable for analysis of histone family members and their PTMs. Almost complete sequence coverage of histone H2A by ProAlanase enables straightforward identification of this protein subfamily members and their PTMs, which none of the other proteases were able to achieve. This significant difference for histone analysis is likely explained by the uniform distribution of prolines and alanines in histones, whereas aspartate and glutamate residues are sporadic leading to few available cleavage sites for Asp-N and Glu-C. Conversely, histones in general and H2A in particular is very rich in arginine and lysine residues resulting in very short tryptic peptides, which is generally difficult to analyze by standard LC–MS/MS. On the contrary, miscleaved ProAlanase-generated histone peptides are typically longer and thus more amenable to mass spectrometric analysis. Finally, because histone preparation from cells typically involves acid-extraction (58), low-pH ProAlanase digestion can readily be implemented in these workflows.

Besides the remarkable impact on protein sequence coverage, the low-pH optimum and short incubation times of ProAlanase also decrease the risk of disulfide bond scrambling during sample preparation. It is, in general, well-accepted that the acidic pH dramatically lowers the chances of disulfide scrambling. For example, in the manuscript by Liu *et al.* (59), disulfide bond scrambling was observed using trypsin both at the pH 7.8 and pH 6.8, whereas no scrambling occurred using pepsin digestion at the pH 1.5. Moreover, we also observed disulfide scrambling during tryptic digestion at the pH 6.8 (37), similarly to the findings done by Lu *et al.* (60), where scrambling took place also at the pH 6.5.

Using ProAlanase at pH 1.5, we observed a higher digestion efficiency of a nonreduced NIST mAb and almost complete coverage of its sequence and disulfide-containing fragments, compared with trypsin. This is most likely because of an acid-induced denaturation of the antibody during ProAlanase digestion, which did not occur during denaturation-free tryptic digestion. This shows the superiority of ProAlanase over trypsin in disulfide bond mapping, achieving better results in shorter time, compared with the standard denaturing workflows for antibody analysis (61). As mentioned above, another protease that is active at pH < 2 and has been previously used in disulfide bond mapping experiments, is pepsin (59, 62). Pepsin, however, possesses much lower digestion specificity than the ProAlanase, which makes ProAlanase more predictable in the data analysis. Taken together, these lines of evidence support the fact that ProAlanase is a powerful tool for efficient low pH disulfide bond mapping.

Finally, we performed a near-complete *de novo* sequencing of affinity purified proline-rich exogenously expressed protein, using a combination of ProAlanase and tryptic peptides. To our knowledge, this is the first example in the field for an exogenously expressed purified protein.

A potential future application for ProAlanase, not covered in this study, is the minimization of artificially-introduced deamidation during sample preparation using low pH digestion (63, 64).

Apart from above described findings, we anticipated an improvement in human proteome coverage when combining peptides generated because of ProAlanase and tryptic digestions of HeLa cell lysate. The results showed an expected increase in proteome coverage, however, less impactful when compared with trypsin+Asp-N or with trypsin+Glu-C peptide combinations (supplemental Fig. S8). These findings support our previous claims on suitability of ProAlanase for targeted, case-specific applications, rather than for proteome-wide context.

Despite its high versatility in proteomic applications, the performance of ProAlanase is limited by a lower digestion efficiency of the protease under reducing conditions and by on average lower identifications, compared with trypsin. The diminished enzymatic activity results in an elevated missed cleavage, producing peptides of atypical lengths and/or charge states that are difficult to analyze by standard RP-LC–MS/MS. The increased miscleavage rates can, potentially, lead to exacerbation of dynamic range issues resulting in multiple overlapping peptides from the same highly-abundant proteins. This, however, can be both problematic for a proteome-wide analysis and beneficial for specific case studies (*e.g.* bone collagen, immunoprecipitated N3ICD, histones). The conventional database search engines are also less optimized for atypical peptide analysis and may face difficulties in tolerating a large search space, introduced by high missed cleavage. To tackle these issues, several actions can be taken. To increase the digestion efficiency further and potentially reduce missed cleavage rates, an on-bead protein aggregation capture (PAC) aided digestion can be used (33). Indeed, PAC digestion was more effective than standard in-solution digestion with 11% more unique peptide sequences fully cleaved (supplemental Fig. S9) in ProAlanase digests of HeLa cell lysate, compared with the standard in-solution digestion.

To increase ProAlanase peptide identifications, the re-scoring of the MaxQuant search results can be done using a new Prosit (65) spectral intensity prediction algorithm. Here, the preliminary results of data re-scoring (supplemental Fig. S10) allowed a ∼20% increase in peptide identifications at 1% FDR in ProAlanase-digested human cell lysate. Finally, as shown by van der Laarse *et al.* (30), the higher spectral matching and consequently higher identification rates can be achieved either by using orthogonal MS/MS fragmentation techniques, such as ETD and EThcD, or by employing alternative data search algorithms.

Summarizing the above mentioned characteristics of ProAlanase, we can conclude that it is multi-functional as well as both alternative and complementary to trypsin. We therefore foresee its high potential usability in various proteomics applications.

## DATA AVAILABILITY

The MS proteomics data have been deposited to the ProteomeXchange Consortium via the PRIDE partner repository with the data set identifiers PXD019039, PXD0121191 and PXD021703. Annotated spectra of post-translationally modified peptides can be visualized using the MS-Viewer in ProteinProspector 6.2.1 Basic (The search key for the saved data sets is **kdgfyaepro**): http://msviewer.ucsf.edu/prospector/cgi-bin/mssearch.cgi?report_title=MS-Viewer&search_key=kdgfyaepro&search_name=msviewer.

## Supplementary Material

supplemental Fig. S5

Supplementary Data 1

Supplementary Data 3

Supplementary Data 2

Supplementary Data 4A

Supplementary Data 4B

Supplementary Data 5

Supplementary Data 6

Supplementary Data 7

Supplementary material

Supplementary Data 9

Supplementary Data 8
