# Supplementary material for: ProAlanase is an Effective Alternative to Trypsin for Proteomics Applications and Disulfide Bond Mapping
Source: Mol Cell Proteomics. 2020 Oct 5;19(12):2139–56. doi: 10.1074/mcp.TIR120.002129 (PMC7710147; doi:10.1074/mcp.TIR120.002129)
Supplement: supplemental Fig. S5 [file TIR120.002129_index.html]

Supplement to ProAlanase is an effective alternative to trypsin for proteomics applications and disulfide bond mapping | Molecular & Cellular Proteomics

## Supplemental Data

- Supplementary Data 1 - Peptide lists for specificity estimation
- Supplementary Data 3 - Peptide lists for sequence motifs
- Supplementary Data 2 - Peptide sequences identified in HeLa, Pleistocene mammoth and N3ICD immunoprecipitate samples.
- Supplementary Data 4A - Protein groups for mammoth bone analysis
- Supplementary Data 4B - Protein groups from analysis of N3ICD
- Supplementary Data 5 - Mammoth peptide lists for maximum missed cleavage estimation
- Supplementary Data 6 - Phosphorylation site analysis of N3ICD
- Supplementary Data 7 - Peptide lists from de novo sequencing of N3ICD
- Supplementary material - Supplementary figures and tables
- Supplementary Data 9 - Protein groups identified in multi-enzymatically digested HeLa cell lysate.
- Supplementary Data 8 - Peptide sequences identified in multi-enzymatically digested HeLa cell lysate.
