# Supplementary material for: ProAlanase is an Effective Alternative to Trypsin for Proteomics Applications and Disulfide Bond Mapping
Source: Mol Cell Proteomics. 2020 Oct 5;19(12):2139–56. doi: 10.1074/mcp.TIR120.002129 (PMC7710147; doi:10.1074/mcp.TIR120.002129)
Supplement: Supplementary material [file 160893_2_supp_604549_qhf682.pdf]

ProAlanase is an effective alternative to trypsin for proteomics applications and disulfide bond mapping

Diana Samodova, Christopher M. Hosfield, Christian N. Cramer, Maria V. Giuli, Enrico Cappellini, Giulia Franciosa, Michael M. Rosenblatt, Christian D. Kelstrup, Jesper V. Olsen

Supplemental Data

- Supplementary material 1 – This file contains peptide sequences identified in HeLa cell lysate digested at the different pH and time points, used to determine the specificity of ProAlanase. The peptide lists are extracted from the MaxQuant "peptides.txt" output files.
- Supplementary material 2 – This file contains peptide sequences identified in HeLa, Pleistocene mammoth and N3ICD immunoprecipitate samples. The peptide lists are extracted from the MaxQuant "evidence.txt" output files and contain the information on precursor charge, m/z, all modifications observed and the corresponding peptide scores.
- Supplementary material 3 – This file contains peptide sequences identified in HeLa cell lysate digested at the different pH and time points. The pH 1.5 2h (optimal digestion conditions) experimental point was used to evaluate sequence context of missed Pro and Ala cleavages. The peptide lists are extracted from the MaxQuant "peptides.txt" output files.

- Supplementary material 4 A – This file contains protein groups identified in Pleistocene mammoth bone sample. The protein lists are extracted from the MaxQuant "proteinGroups.txt" output files and contain the information on protein accession numbers, unique peptide count assigned to each protein and protein sequence coverage in %.
- Supplementary material 4 B – This file contains protein groups identified in N3ICD immunoprecipitate. The protein lists are extracted from the MaxQuant "proteinGroups.txt" output files and contain the information on protein accession numbers, unique peptide count assigned to each protein and protein sequence coverage in %.
- Supplementary material 5 – This file contains unique peptide sequences identified in Pleistocene mammoth bone sample, used to determine the optimal maximum missed cleavage. The peptide lists are extracted from the MaxQuant "peptides.txt" output files.
- Supplementary material 6 – This file contains phosphorylation sites localized in N3ICD immunoprecipitate. The phosphosite lists are extracted from the MaxQuant Phospho (STY)Sites.txt output files and contain the information on phosphosite localization probability, score and intensity of phosphorylated peptides.
- Supplementary material 7 – This file contains *de novo* peptide sequences identified in N3ICD immunoprecipitate. *De novo* peptide lists are extracted from the PEAKS "de novo peptides.csv" output files and contain the information on precursor charge, m/z, all modifications observed and the corresponding Denovo scores and ALC confidence.
- Supplementary material 8 – This file contains unique modified peptide sequences identified in multi-enzymatic digests of HeLa cell lysate for the data searched with

additional histone-specific variable modifications. The modified peptide lists are extracted from the MaxQuant "evidence.txt" output files and contain the information on precursor charge, m/z, all modifications observed and the corresponding peptide scores.

- Supplementary material 9 – This file contains protein groups identified in multi-enzymatic digests of the HeLa cell lysate. The protein lists are extracted from the MaxQuant "proteinGroups.txt" output files and contain the information on protein accession numbers, unique peptide count assigned to each protein and protein sequence coverage in %.
- Supplementary table S1 – This table contains members of the different histone subfamilies, identified in multi-enzymatic digests of HeLa cell lysate. The table reflects protein sequence coverage, obtained using ProAlanase, tryptic, Glu-C and Asp-N-generated peptides.
- Supplementary table S2 – This table contains proteins identified in Pleistocene mammoth bone. The table reflects protein sequence coverage, obtained by ProAlanase and tryptic-peptides and their combination.
- Supplementary table S3 - This table displays N3ICD protein coverage by high-confidence *de novo* peptides generated as a result of multi-enzymatic digestions.
- Supplementary figures and legends – This file contains supplementary figures and their legends.

Supplementary Tables

Table S1 Histone protein coverage by different proteases.

Family	Protein ID	Member	Average Seq. coverage %, ProAlanase	Average Seq. coverage %, Trypsin	Average Seq. coverage %, Glu-C	Average Seq. coverage %, Asp-N
H1	P07305	Histone H1.0	-	10	-	-
	Q02539	Histone H1.1	11	33	5	-
	P16403	Histone H1.2	39	-	21	-
	P16402	Histone H1.3	36	-	-	-
	P10412	Histone H1.4	13	36	-	-
	P16401	Histone H1.5	9	35	20	-
	Q92522	Histone H1x	-	21	-	8.9
H2A	P0C0S8	Histone H2A type 1	91	-	30	-
	P20671	Histone H2A type 1-D	91	-	-	-
	Q6FI13	Histone H2A type 2-A	91	46	30	-
	Q93077	Histone H2A type 1-C	91	46	-	-
	Q16777	Histone H2A type 2-C	91	-	-	-
	Q96KK5	Histone H2A type 1-H	91	-	-	-
	Q99878	Histone H2A type 1-J	91	-	-	-
	Q8IUE6	Histone H2A type 2-B	73	-	-	14
	Q9BTM1	Histone H2A.J	71	-	-	-
	P16104	Histone H2AX	56	-	-	-
	P0C0S5	Histone H2A.Z	43	-	-	-
	Q71UI9	Histone H2A.V	14	31	-	-
H2B	P33778	Histone H2B type 1-B	-	-	39	14
	Q8N257	Histone H2B type 3-B	-	-	39	-
	P58876	Histone H2B type 1-D	57	-	-	-
	Q96A08	Histone H2B type 1-A	22	-	-	-
	O60814	Histone H2B type 1-K	54	-	39	-
	P06899	Histone H2B type 1-J	-	81	-	-
	Q16778	Histone H2B type 2-E	-	81	-	-
	P62807	Histone H2B type 1-C/E/F/G/I	-	81	-	-
H3B	P68431	Histone H3.1	59	75	-	-
	Q71DI3	Histone H3.2	59	69	-	-
	P84243	Histone H3.3	50	60	-	-
H4B	P62805	Histone H4	32	81	49	34

*Only leading proteins were considered in each protein group. The protein had to be identified in at least 3 experimental replicates for ProAlanase and tryptic samples (total n=4 for each protease) and in at least 2 replicates for Glu-C and Asp-N (total n=3).

Table S2 Proteins identified in Pleistocene mammoth bone.

Protein ID	Gene name	Protein name	Species-specific amino acid substitutions	Sequence coverage (%), Trypsin	Sequence coverage (%), ProAlanase	Sequence coverage (%), Trypsin+ProAlanase
G3TIC0	COL1A2	Collagen type I alpha 2 chain	-	78	79	83
G3SSE0; G3UE48	COL1A1	Collagen type I alpha 1 chain	-	73	79	82
G3TLG4	APOA4	Apolipoprotein A4	+	72	32	76
G3T019	AHSG	Alpha 2-HS glycoprotein or Fetuin-A	+	51	63	75
G3TEY6	SERPINF1	Serpin family F member 1	+	66	49	75
G3SMX8	ALB	Albumin	+	59	41	72
G3T3V1	CHAD	Chondroadherin	-	67	40	71
G3TMK8	SERPINA10	Serpin family A member 10	+	64	50	69
G3SQ12	BGN	Biglycan	-	60	41	63
G3TJT8	SPARC	Secreted protein acidic and cysteine rich	-	53	38	62
G3SU45	SERPINC1	Serpin family C member 1	+	59	8	62
G3TS89	COL5A2	Collagen type V alpha 2 chain	-	45	31	57
G3T5I1	F2	Prothrombin (Coagulation factor II)	+	44	33	52
G3SYR8	DCN	Decorin	-	36	22	51
G3TI02	PCOLCE	Procollagen C-endopeptidase enhancer	+	42	20	49
G3U8J5	N/A	Uncharacterized protein	-	23	34	49
G3SYR5	LUM	Lumican	+	34	20	48
G3TER5	SPP2	Secreted phosphoprotein 2	+	38	20	47
G3SQ01	OMD	Osteomodulin	+	38	21	47
G3T147	OLFML3	Olfactomedin like 3	-	30	39	41
G3T9F6	COL11A1	Collagen type XI alpha 1 chain	-	26	17	38
G3TMX5	N/A	SMB domain-containing protein	-	22	26	38
G3U7X8	COL5A1	Collagen type V alpha 1 chain	-	31	24	36
G3TD13	APOE	Apolipoprotein E	+	35	-	36
G3U8A3	CLEC3B	C-type lectin domain family 3 member B	-	23	18	34
G3TV86	F9	Coagulation factor IX	-	31	19	32
G3TZT8	C8G	Complement C8 gamma chain	+	32	-	32
G3TQ58	C9	Complement C9	+	31	-	31
G3UNP6	N/A	Uncharacterized protein	-	16	25	31
G3TSP3	N/A	Uncharacterized protein	-	18	-	30

*Only proteins with sequence coverage ≥ 30% are displayed. Protein sequence coverage is compared between different proteases (trypsin and ProAlanase) and different data search strategies (tryptic fractions merged for 2 replicares, ProAlanase fractions merged for 2 replicates and tryptic+ProAlanase fractions merged for 2 replicates). Only leading proteins from each protein group are displayed.

Table S3 N3ICD sequence coverage by multi-enzymatic *de novo* peptides.

Protease	N3ICD sequence coverage, %
Trypsin+ProAlanase+GluC+AspN	90
Trypsin+ProAlanase+AspN	90
Trypsin+ProAlanase	89
Trypsin+ProAlanase+GluC	89
ProAlanase+GluC+AspN	81
ProAlanase+AspN	80
Trypsin+AspN	75
Trypsin+GluC+AspN	75
Trypsin+GluC	74
ProAlanase+GluC	60
GluC+AspN	52

*Peptides from 2 biological replicates were concatenated for each protease.

.

Supplementary Figures

Supplementary Figure 1

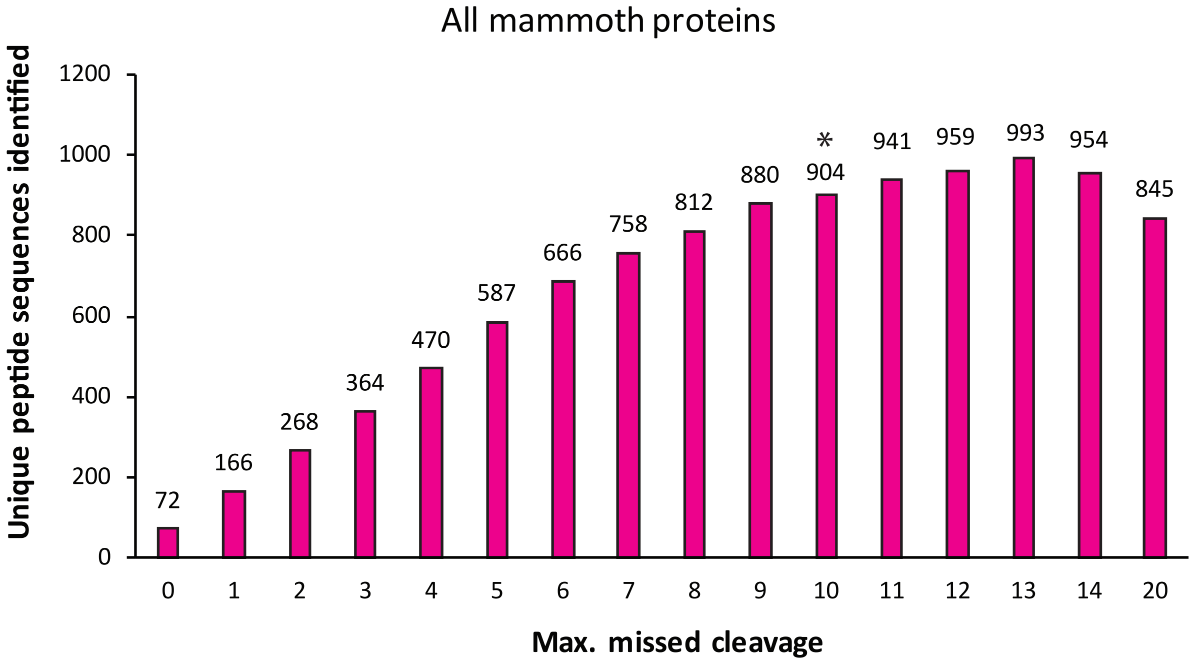

Supplementary Figure 1 Distribution of unique mammoth peptides over the maximum missed cleavage. Unique peptide identifications in ProAlanase-digested Pleistocene mammoth bone pellet (n = 1), observed at the different maximum missed cleavage values. The highest peptide identifications are observed, searching the data with 13 missed cleavages. The loss of true positive identifications is observed, when the maximum missed cleavage value is increased to 14. The maximum missed cleavage of 10 amino acids (marked with *) was considered to be optimal, as a compromise solution between the search space size and the number of unique peptide sequences identified.

Supplementary Figure 2

a)

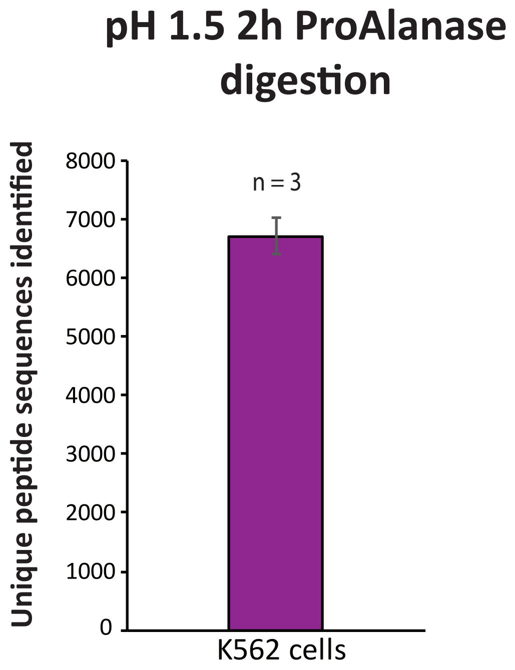

b)

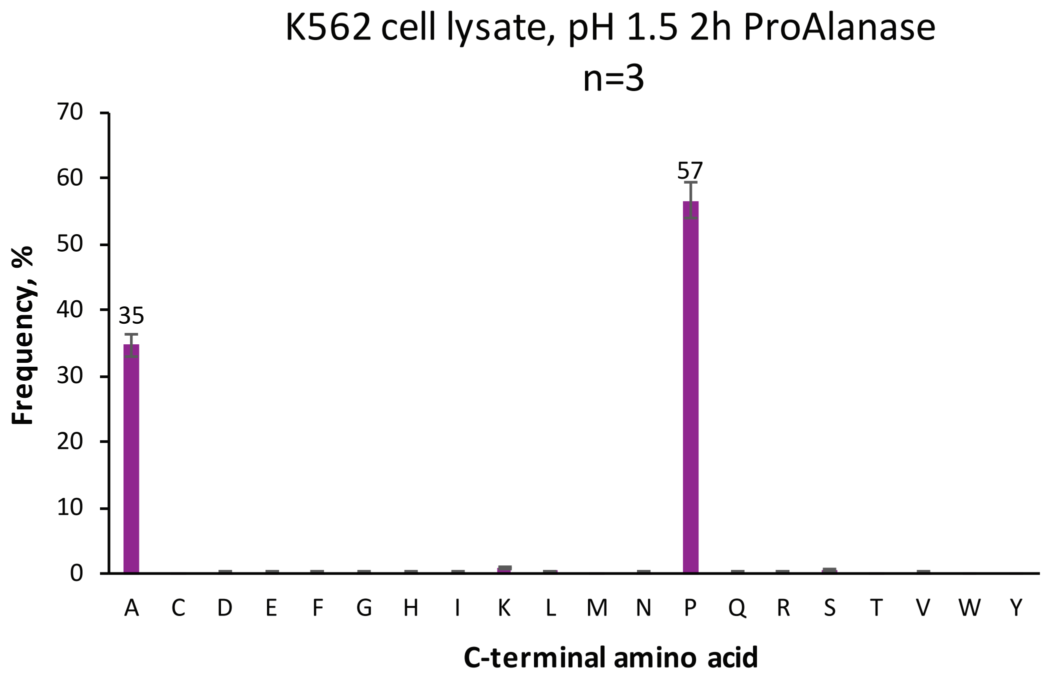

Supplementary Figure 2 Unique peptide sequences identified (a) in K562 cell lysate (n=3), digested at the optimal ProAlanase digestion conditions (pH 1.5 2h). Specificity plot (b) reflecting C-terminal amino acid frequency in ProAlanase-digested K562 cell lysates (n = 3) at the optimal pH and time of digestion**.**

Supplementary Figure 3

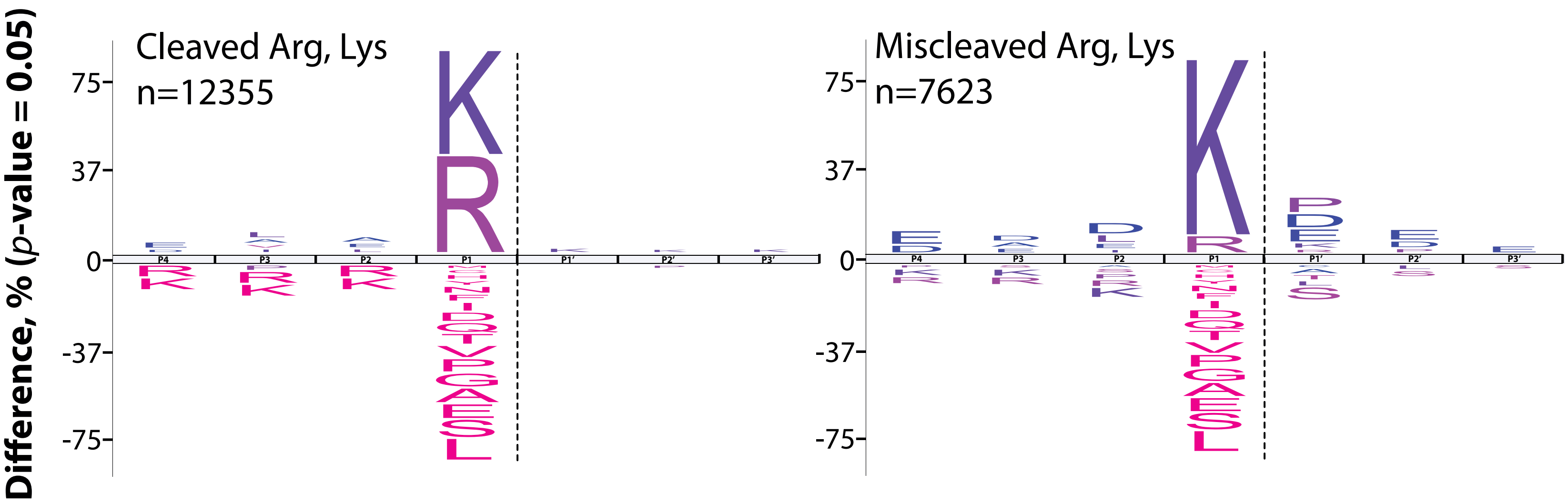

Supplementary Figure 3 Characterization of missed cleavage patterns in trypsin-digested HeLa cell lysate. Frequency distribution plots of cleavage sites identified in human proteome samples, digested with trypsin. The unique peptide sequences from the two experimental replicates were concatenated to evaluate sequence context of missed arginine and lysine cleavages. Sequences were aligned at cleavage sites between P1 and P1′. The significant differences in amino acid occurrence are compared to the natural abundance in human proteome ($P < 0.05$).

Supplementary Figure 4

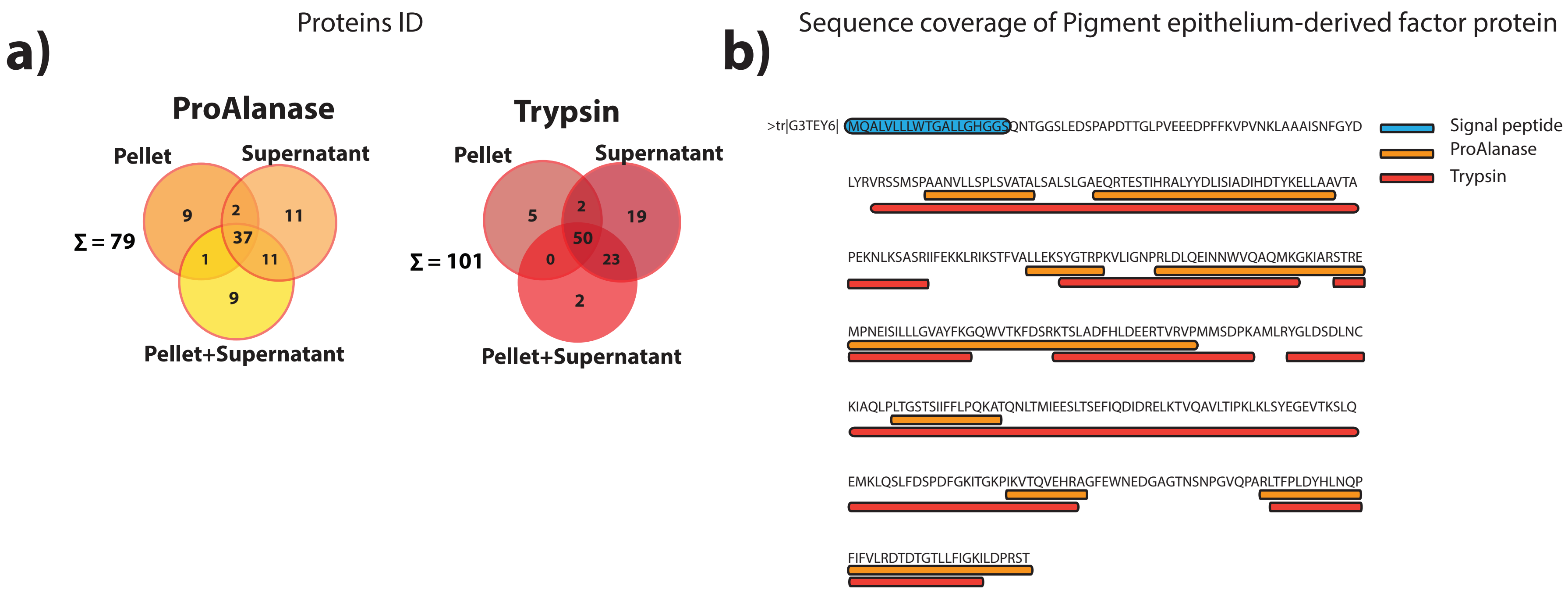

Supplementary Figure 4 Additional data on Pleistocene mammoth bone palaeoproteomic characterization, using ProAlanase and trypsin.
Venn Diagrams (a) displaying shared and complementary protein groups in ProAlanase- and trypsin-digested Pleistocene mammoth bone sample fractions. The overlap was done based on the proteins concatenated from the 2 experimental replicates. Only leading proteins in each protein group were used to do the overlap. Sequence coverage of *Loxodonta africana* Pigment epithelium-derived factor protein (b), complemented by ProAlanase and tryptic peptides.

Supplementary Figure 5 A

Fetuin-A multiple sequence alignment, part 1

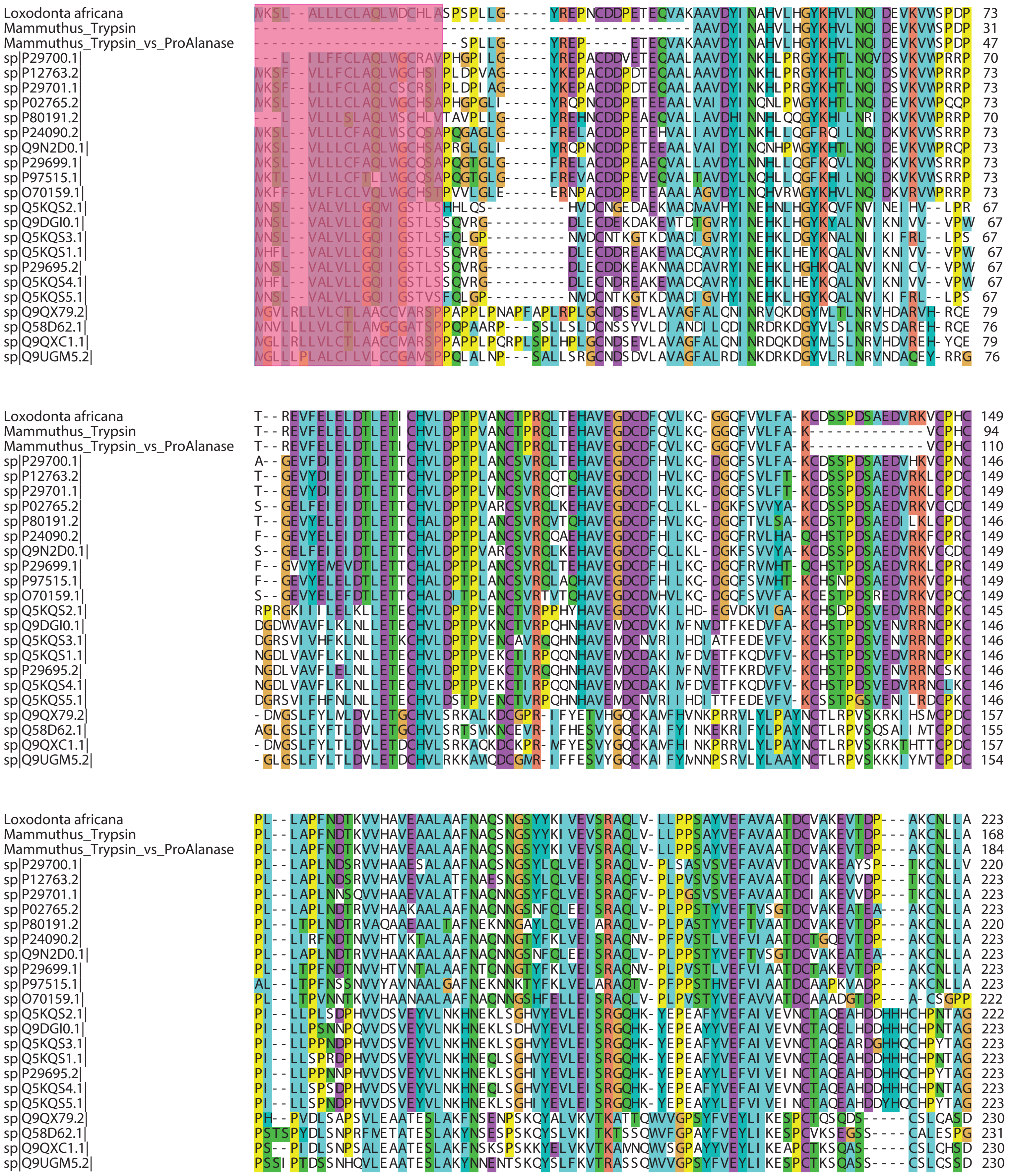

Supplementary Figure 5 B

Fetuin-A multiple sequence alignment, part 2

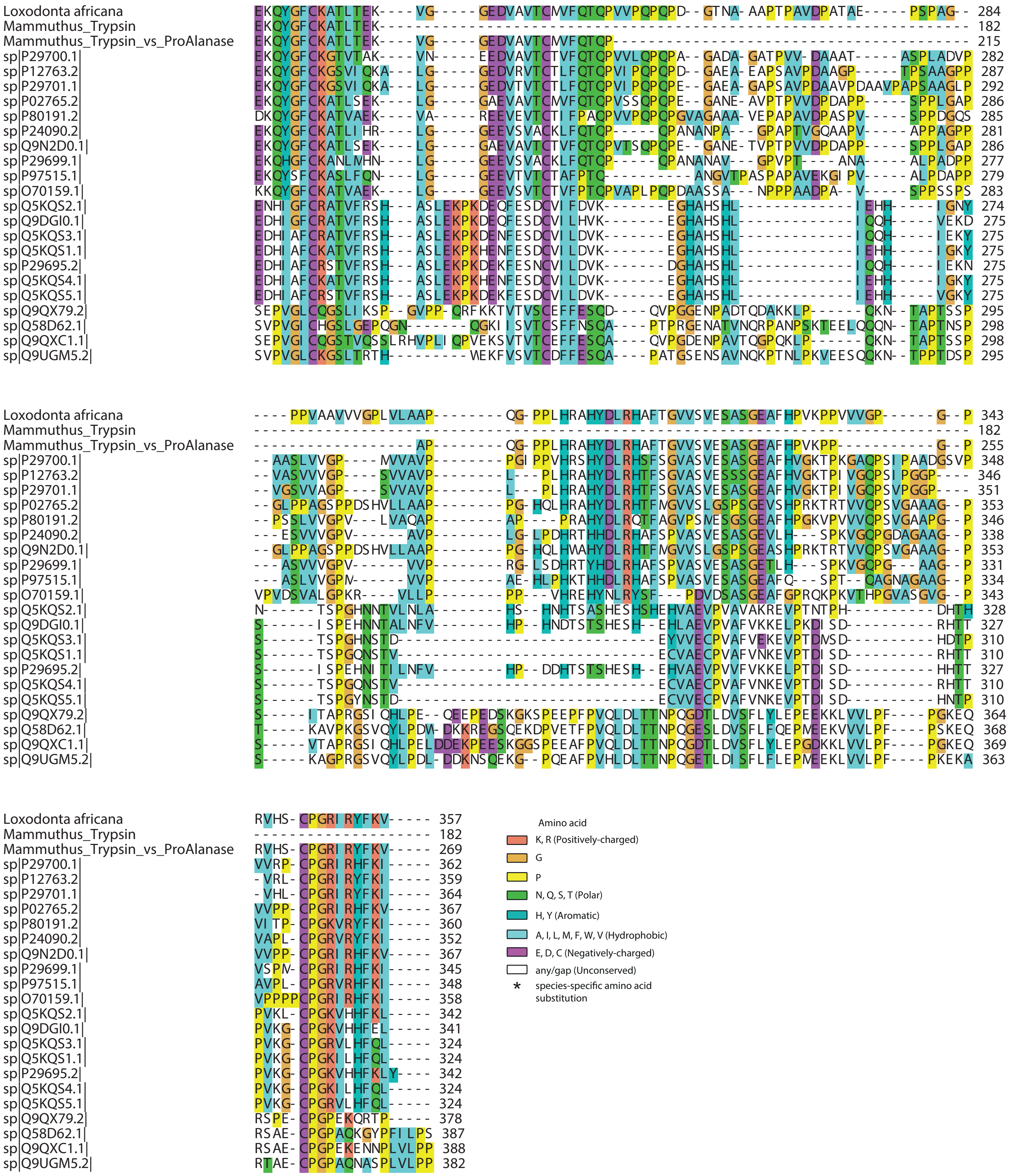

Supplementary Figure 5 Complete multiple sequence alignment of fetuin-A across different species. Multiple sequence alignment of fetuin-A protein sequences between Pleistocene mammoth fetuin and the other mammalian species with BLASTp identity ≥ 50%. The signal peptide is marked with pink rectangle. The alignment was performed using ClustalX2. All proteinaceous fractions of Pleistocene mammoth bone extract were merged for the 2 replicates in a single MaxQuant search by protease (ProAlanase/trypsin) and in combination (ProAlanase+ trypsin).

Supplementary Figure 6

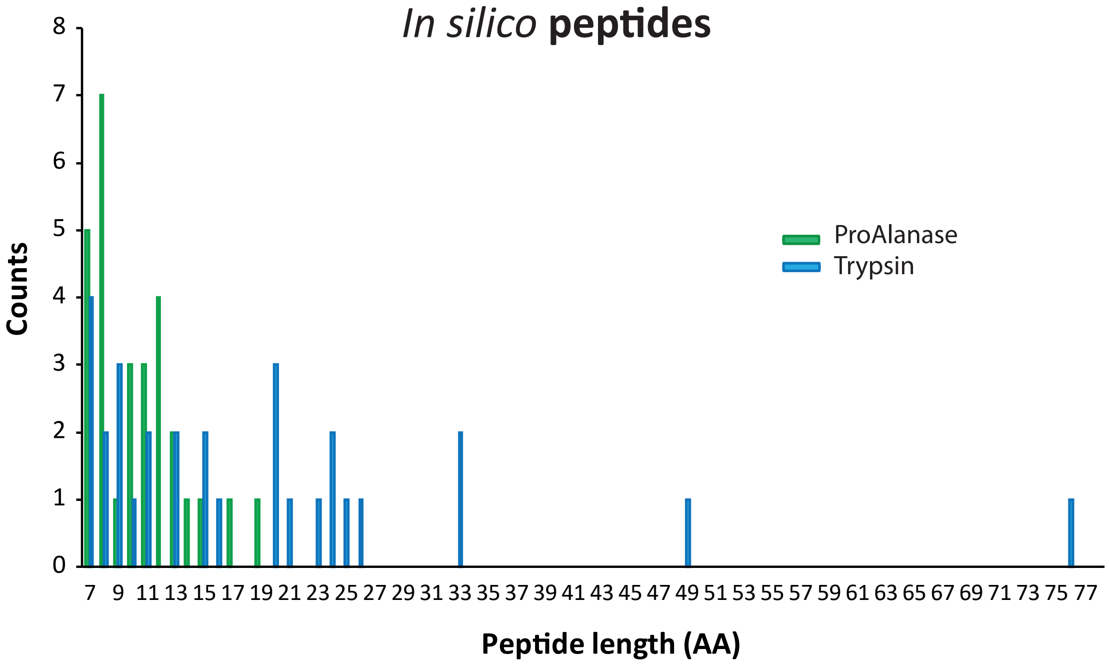

Supplementary Figure 6 *In silico* **digestion of Notch3 intracellular domain protein (N3ICD).**
An *in silico* digestion plot showing the distribution of N3ICD peptide counts over the different lengths, obtained from ProAlanase and tryptic *in silico* digestion. Only peptides with 0 missed cleavages and length ≥ 7 are displayed.

Supplementary Figure 7

Disulfide bond identification workflow

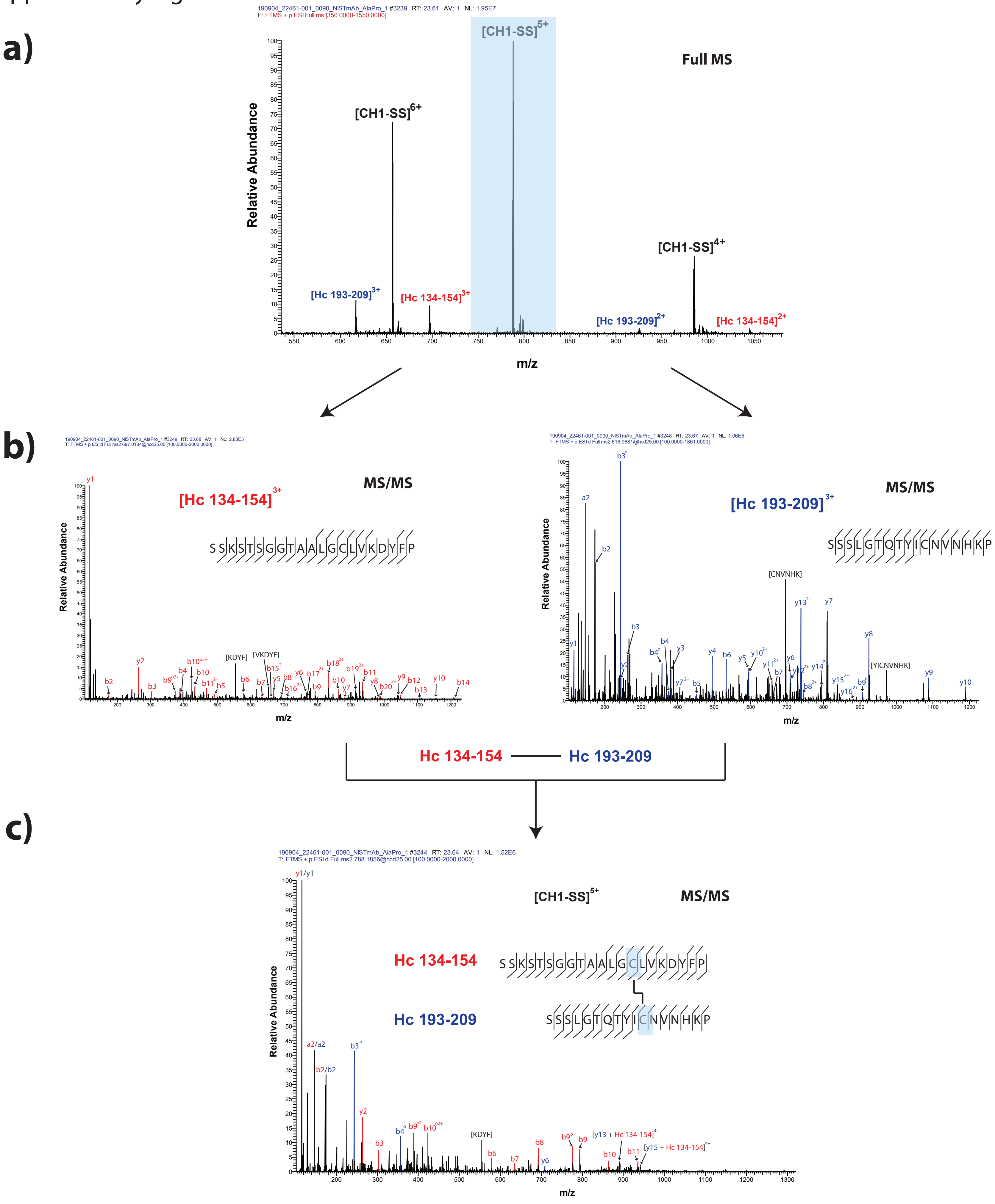

Supplementary Figure 7 Disulfide bond identification workflow.
MS1 spectrum (a) showing partial reduction of CH1-SS disulfide bond. MS2 spectra (b) displaying the fragmentaion of CH1-SS-composing reduced peptides. MS2 spectrum (c) of constituting reduced peptides, linked via a disulfide bond.

Supplementary Figure 8

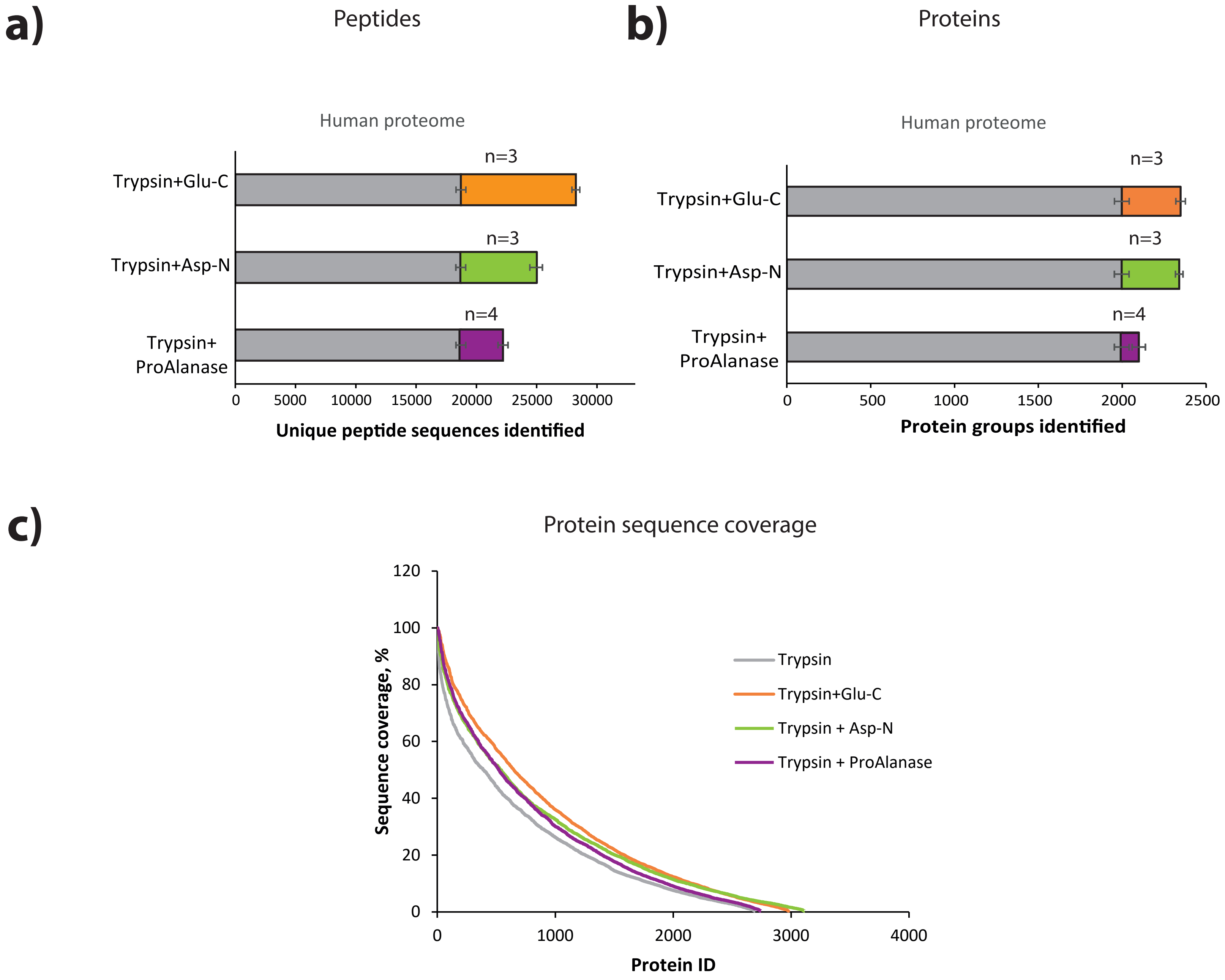

Supplementary Figure 8 Human proteome coverage by the different combinations of peptides obtained from multi-enzymatic digests of HeLa cell lysate.
Bar charts (a and b) displaying an increase in average unique peptide (a) and protein group (b) identifications, when combining tryptic and Glu-C/Asp-N/ProAlanase peptides obtained from the digests of HeLa cell lysate (n=3 or 4 respectively).
Sequence coverage distribution (c) over the range of identified human proteins using different combinations of peptides obtained from tryptic (n=2), ProAlanase (n=2), Asp-N (n=2) and Glu-C (n=2) digests of HeLa cell lysate.

Supplementary Figure 9

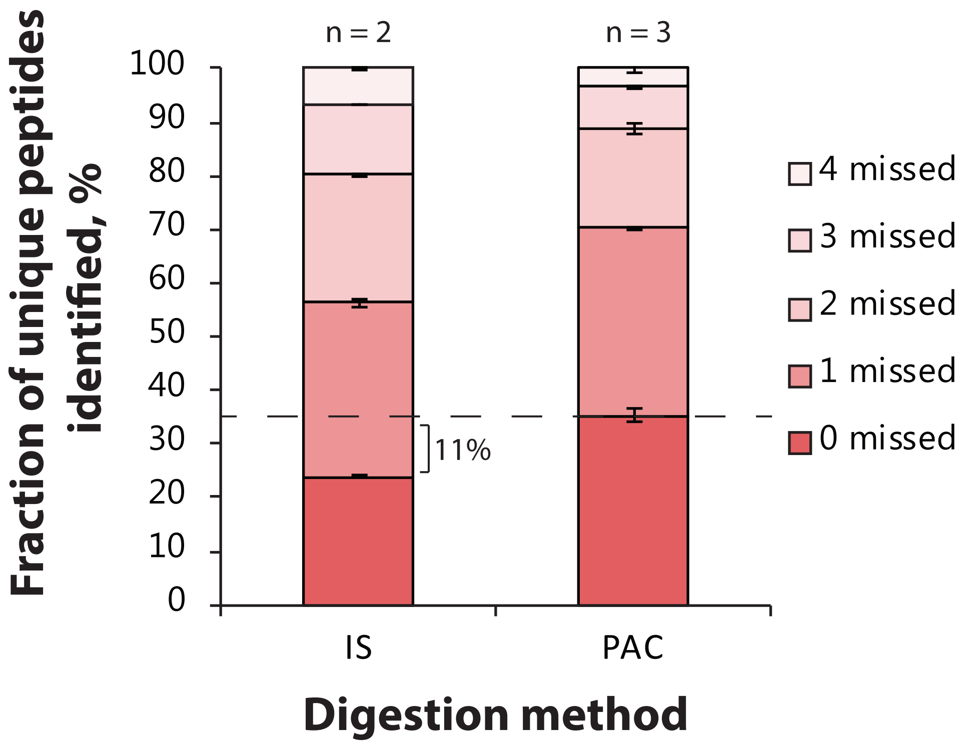

Supplementary Figure 9 Fraction of unique peptides identified in ProAlanase digests of HeLa cell lysates carrying 0, 1, 2, 3 or 4 missed cleavage sites, using enzyme-specific settings for peptide-to-sequence matching. The comparison is done for the two different digestion methods - in-solution (IS, n = 2) and protein aggregation capture (PAC, n = 3) workflows.

Supplementary Figure 10

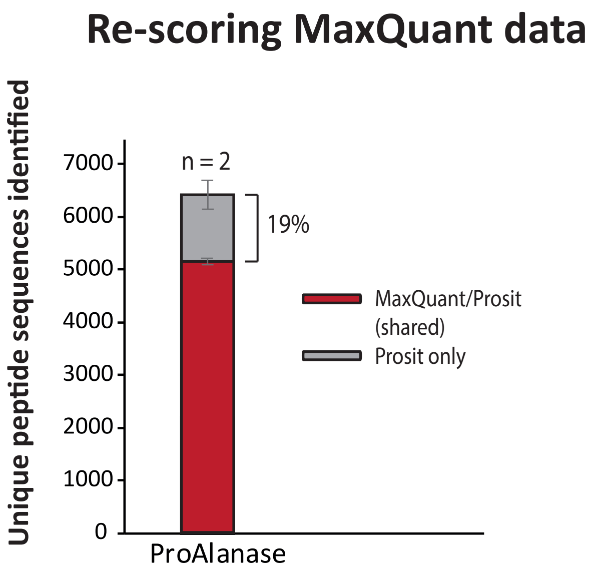

Supplementary Figure 10 Re-scoring MaxQuant search results using Prosit spectral intensity prediction algorithm. Average number of unique peptide identifications in ProAlanase-digested human proteome samples (n = 2). The red part of the barplot corresponds to the shared peptide sequences, identified both using the MaxQuant Andromeda set of scores at 1% peptide level FDR and the Prosit peptide scores at the same FDR level. The grey part of the barplot corresponds to the peptides identified by Prosit only, at 1% peptide FDR.
